# Citrus tristeza virus p20 suppresses antiviral RNA silencing by co-opting autophagy-related protein 8 to mediate the autophagic degradation of SGS3

**DOI:** 10.1371/journal.ppat.1012960

**Published:** 2025-02-24

**Authors:** Yongle Zhang, Zuokun Yang, Zhe Zhang, Guoping Wang, Xiang-Dong Li, Ni Hong

**Affiliations:** 1 Key Lab of Plant Pathology of Hubei Province, College of Plant Science and Technology, Huazhong Agricultural University, Wuhan, China; 2 National Key Laboratory for Germplasm Innovation & Utilization of Horticultural Crop, Huazhong Agricultural University, Wuhan, China; 3 Department of Plant Pathology, Shandong Provincial Key Laboratory of Agricultural Microbiology, College of Plant Protection, Shandong Agricultural University, Tai’an, Shandong, China; 4 Institute of Plant Protection, Shandong Academy of Agricultural Sciences, Ji’nan, Shandong, China; China Agricultural University, CHINA

## Abstract

Viruses exploit autophagy to degrade host immune components for their successful infection. However, how viral factors sequester the autophagic substrates into autophagosomes remains largely unknown. In this study, we showed that p20 protein, a viral suppressor of RNA silencing (VSR) encoded by citrus tristeza virus (CTV), mediated autophagic degradation of SUPPRESSOR OF GENE SILENCING 3 (SGS3), a plant-specific RNA-binding protein that is pivotal in antiviral RNA silencing. CTV infection activated autophagy, and the overexpression of p20 was sufficient to induce autophagy. Silencing of autophagy-related genes *NbATG5* and *NbATG7* attenuated CTV infection in *Nicotiana benthamiana* plants. In contrast, knockdown of the autophagy negative-regulated genes *NbGAPCs* led to virus accumulation, indicating the proviral role of autophagy in CTV infection. Further investigation found that p20 interacted with autophagy-related protein ATG8 through two ATG8-interacting motifs (AIMs) and sequestered SGS3 into autophagosomes by forming the ATG8-p20-SGS3 ternary complex. The mutations of the two AIMs in p20 (p20^mAIM1^ and p20^mAIM5^) abolished the interaction of p20 with ATG8, resulting in the deficiency of autophagy induction, SGS3 degradation, and VSR activity. Consistently, *N. benthamiana* plants infected with mutated CTV^mAIM1^ and CTV^mAIM5^ showed milder symptoms and decreased viral accumulation. Taken together, this study uncovers the molecular mechanism underlying how a VSR mediates the interplay between RNA silencing and autophagy to enhance the infection of a closterovirus.

## Author summary

Viruses manipulate autophagy to suppress host immune response for their infection. However, the mechanisms by which viral factors mediate the autophagic degradation of host components remain largely unexplored. Here, we showed that citrus tristeza virus (CTV) p20 protein mediated autophagic degradation of SUPPRESSOR OF GENE SILENCING 3 (SGS3), a core component in antiviral RNA silencing. CTV infection and the p20 expression could induce autophagy, while the activated autophagy promoted CTV infection. Significantly, p20 interacted with the autophagy-related protein ATG8 and sequestered SGS3 into autophagosomes by forming ATG8-p20-SGS3 ternary complexes. Furthermore, we discovered that two ATG8-interacting motifs (AIMs) responsible for the interaction between p20 and ATG8 were crucial for p20-mediated autophagy activation and RNA silencing suppression. These data suggest that a plant virus protein plays a selective autophagy receptor-like role in hijacking RNA silencing components into autophagosomes for degradation.

## Introduction

Citrus tristeza virus (CTV), which belongs to the genus *Closterovirus* in the family *Closteroviridae*, is one of the most destructive pathogens of citrus plants worldwide [1,2]. CTV possesses a 19.3 kb positive single-stranded genomic RNA, containing 12 open reading frames (ORFs) flanked by 5′- and 3′-untranslated regions [1,3]. ORFs 1a and 1b encode replication-related proteins expressed from the genomic RNA. ORFs 2–11 encode proteins expressed via 3′-coterminal subgenomic RNAs, including p33, p6, p65, p61, minor coat protein (CPm), coat protein (CP), p18, p13, p20, and p23. The p20 is an important pathogenic factor. The protein co-localizes with p33 and accumulates as small inclusions in CTV-infected cells [4–6]. p20 is necessary for the virus systemic infection and is a viral effector that triggers reactive oxygen species burst in *Nicotiana benthamiana* [7–9]. Moreover, p20 is a viral suppressor of RNA silencing (VSR) that inhibits intercellular and intracellular silencing of plants [10]. Nevertheless, the strategy of the viral proteins to counter RNA silencing is rarely explored for the viruses in the genus *Closterovirus*.

Autophagy is a highly conservative degradation and self-renewal system in eukaryotic organisms [11]. In plants, autophagy delivers cellular components, including damaged organelles, macromolecules, and selected proteins, to vacuoles for degradation, thereby playing essential roles in plant development and response to various biotic and abiotic stresses [12]. Selective autophagy is a distinct type of autophagy that relies on selective autophagy receptors (SARs) to specifically target particular cargoes [12,13]. SARs function as linkages between cargoes and autophagy-related protein ATG8, essential for delivering autophagy substrates into autophagosomes and triggering phagophore formation [13,14]. Usually, SARs contain ATG8-interaciting motifs (AIMs) or ubiquitin-interacting motifs to bind with ATG8/LC3 [15,16]. Increasing evidence has indicated that selective autophagy plays versatile functions in plant-virus interaction [17]. In most cases, SAR-mediated selective autophagy plays a critical role in plant defense against virus infection. The well-known SAR neighbor of BRCA1 gene 1 (NBR1) functions in the targeted degradation of cauliflower mosaic virus (CaMV) particles and the viral capsid proteins [18]. The autophagy core component ATG6/Beclin1 interacts with turnip mosaic virus (TuMV) nuclear inclusion protein b (NIb) and ATG8a to mediate the degradation of NIb [19]. On the other hand, plant viruses have evolved to avoid antiviral autophagy for their infection. Barley stripe mosaic virus γa and γb proteins suppress antiviral autophagy by blocking vacuolar acidification and interfering with ATG7-ATG8 interaction, respectively [20,21]. Plant viruses can also subvert autophagy to degrade antiviral factors for its benefit. For example, RSV movement protein (MP), NSvc4, facilitates viral movement by mediating the autophagic degradation of remorin, a negative regulator for RSV movement [22]. It is largely unknown whether autophagy is involved in plant-closterovirus interaction.

RNA silencing is a conserved antiviral mechanism in plants [23]. For post-transcriptional gene silencing, the RNA silencing is initiated by double-stranded RNAs (dsRNAs) produced during virus replication. Dicer-like enzymes (DCLs) recognize and cleave the dsRNAs into virus-derived small interference RNAs (vsiRNAs). Subsequently, these vsiRNAs are integrated into the RNA-induced silencing complexes to direct the degradation or translational repression of viral genomic RNAs [23,24]. In plants, the RNA-binding protein SUPPRESSOR OF GENE SILENCING 3 (SGS3) cooperates with RNA-dependent RNA polymerase 6 (RDR6) to generate secondary siRNAs for RNA silencing signal amplification [25–27]. To establish successful infection, plant viruses have evolved multiple strategies to counteract antiviral silencing [28]. One of the mechanisms is to degrade core proteins of RNA silencing via viral proteins or virus-co-opted host factors. For example, in the presence of the P0 protein of turnip yellows virus, AGO1 co-localizes with ATG8, and the protein content of AGO1 increased after treatment with autophagy inhibitors [29]. Two ATG8-interacting proteins, ATI1 and ATI2, are involved in the P0-mediated autophagic degradation of AGO1 by mediating the delivery of AGO1 from the endoplasmic reticulum to the vacuole [16]. Previous works have revealed that the virus-induced plant endogenous protein NbCaM and small peptide VISP1 promote autophagic degradation of SGS3 [30,31]. Some VSRs, including TuMV VPg, tomato zonate spot virus (TZSV) NSs, and rice stripe mosaic virus (RSMV) p4, mediate the degradation of SGS3 via both autophagy and ubiquitination pathways [32–34]. VISP1 is a virus co-opted SAR to target SGS3/RDR6 bodies for autophagic degradation by interacting with ATG8 [31]. However, the underlying molecular mechanism of viral protein-mediated SGS3 autophagic degradation remains to be elucidated.

In this study, we revealed that p20, a VSR of the closterovirus CTV, played a SAR-like role in regulating the turnover of the SGS3 protein. The p20 induced host autophagy and served as a bridge to link SGS3 to the essential autophagy protein ATG8, then translocating SGS3 into autophagosomes for degradation to suppress antiviral RNA silencing. Our results elucidated how a viral protein carried a plant RNA silencing component into autophagosomes to curb the plant antiviral response.

## Results

### CTV p20 mediates the autophagic degradation of SGS3

To understand the RNA silencing pathway impaired by CTV p20, we performed the sRNA sequencing by co-agroinfiltrated GFP with p20-Flag in transgenic *N. benthamiana* 16c plants, which are widely used in RNA silencing assay [35]. Sequencing results revealed that the relative abundance of *GFP* siRNAs in p20-Flag-infiltrated patches was approximately 7.6-fold lower than that in empty vector (EV)-infiltrated control patches (S1A Fig). However, the expression of p20-Flag did not affect the sizes, distribution profiles, and the dominant proportion of uridine (U) at the 5′-terminal of *GFP* siRNAs (S1B–S1D Fig). These results suggest that p20 mainly inhibits the siRNA amplification.

**Fig 1:**
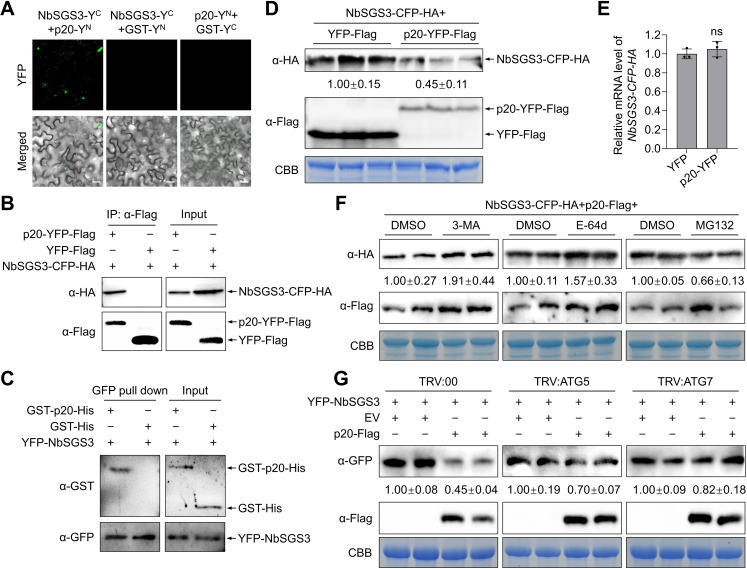
CTV p20 interacts with NbSGS3 and mediates autophagic degradation of NbSGS3. (A) Bimolecular fluorescence complementation assay of the interaction between p20 and NbSGS3. Scale bars, 20 µm. (B) Co-immunoprecipitation assay of the interaction between p20 and NbSGS3 in *Nicotiana benthamiana* leaves. (C) Pull-down assay for the interaction of p20 with NbSGS3. YFP-NbSGS3 was agroinfiltrated into plant leaves. The total protein was precipitated by anti-GFP-trapped agarose, followed by adding purified GST-p20 or GST. The GFP-bound precipitates were analyzed by immunoblotting assays. (D, E) The effect of the p20-YFP-Flag expression on the accumulation of NbSGS3-CFP-HA in *N. benthamiana* leaves. The protein (D) and transcription (E) levels of NbSGS3-CFP-HA were measured by immunoblotting and RT-qPCR assay. In qPCR assay, the *NbActin* gene served as an internal control. Values represent means ± SD from three independent experiments. Significant differences were identified using a one-tailed Student’s *t*-test (ns, no significance). (F) The effect of the autophagy inhibitors 3-MA, E-64d, and proteasome inhibitor MG132 on p20-mediated degradation of NbSGS3. The leaves co-agroinfiltrated with p20-Flag and NbSGS3-CFP-HA were treated with 3-MA (10 mM), E-64d (100 μM), MG132 (100 μM) or DMSO at 48 h post-inoculation and collected at 12 h after the treatments. The accumulation level of NbSGS3-CFP-HA protein was measured by immunoblotting assay. (G) The effect of *NbATG5*- and *NbATG7*-silencing on the p20-mediated degradation of NbSGS3. YFP-NbSGS3 was co-agroinfiltrated with p20-Flag or EV into *NbATG5*-silenced, *NbATG7*-silenced, and non-silenced *N. benthamiana* leaves, respectively. The accumulation level of YFP-NbSGS3 protein was measured by immunoblotting assay. In (D), (F), and (G), coomassie blue staining (CBB) of the Rubisco large subunit was used as a protein loading control, and the band intensities were calculated by ImageJ and normalized to the loading control.

Considering the vital role of SGS3 in siRNA synthesis [25,36], we validated the interaction between p20 and NbSGS3 by bimolecular fluorescence complementation (BiFC) assays. The expression of proteins in the BiFC assay was confirmed by immunoblotting analysis (S2A Fig). Results showed that the co-expression of p20-Y^N^ with NbSGS3-Y^C^ produced YFP signals as small dots and some larger aggregates in the cytoplasm, while the leaves expressing control combinations did not show any YFP signal (Fig 1A). Further co-immunoprecipitation (Co-IP) and pull-down assays confirmed that the p20 specifically interacted with NbSGS3. In the Co-IP test, NbSGS3-CFP-HA fusion protein co-immunoprecipitated with p20-YFP-Flag but not with the negative control YFP-Flag (Fig 1B). In the pull-down test, YFP-NbSGS3 interacted directly with GST-p20, but not the GST control (Fig 1C).

In the Co-IP assays, we noticed that the accumulation level of NbSGS3-CFP-HA decreased in p20-YFP-Flag-expressing leaves (Fig 1B). Then, we validated the effect of p20 on the accumulation level of NbSGS3 by co-agroinfiltrating NbSGS3-CFP-HA with p20-YFP-Flag or YFP-Flag in *N. benthamiana* leaves. Immunoblotting assay results showed that the expression of p20-YFP-Flag significantly reduced the accumulation level of NbSGS3-CFP-HA protein (Fig 1D). However, the expression of p20-YFP-Flag did not change the mRNA level of *NbSGS3-CFP-HA* at three days post-inoculation (dpi) as tested by the reverse transcription quantitative PCR (RT-qPCR) assays (Fig 1E). The results indicate that p20 reduces the stability of NbSGS3. As SGS3 and RDR6 form siRNA bodies for siRNA amplification [26], we examined whether p20 reduced the stability of NbRDR6 by co-expressing NbRDR6-HA with p20-YFP-Flag or YFP-Flag. The results revealed that the protein and transcription levels of NbRDR6-HA were not affected by p20-YFP expression (S3 Fig).

Next, we determined which protein degradation system was responsible for the p20-mediated degradation of NbSGS3. The fusion protein NbSGS3-CFP-HA was co-expressed with p20-Flag in *N. benthamiana* leaves. At 48 hpi, the infiltrated patches were treated individually with dimethyl sulfoxide (DMSO, control), 3-MA (autophagy inhibitor), E-64d (autophagy inhibitor), or MG132 (proteasome inhibitor). Immunoblotting results showed that the accumulation level of NbSGS3-CFP-HA was notably higher in 3-MA- and E-64d-treated leaf patches than that in DMSO-treated leaf patches (Fig 1F). By contrast, upon treatment with MG132, the accumulation level of NbSGS3-CFP-HA reduced to about 0.66-fold of DMSO-treated patches (Fig 1F). Meanwhile, the *NbSGS3-CFP-HA* mRNA level was not significantly affected by any chemical inhibitor (S4A Fig). These results indicate that p20 mediates the NbSGS3 degradation through the autophagy pathway.

To further evaluate the role of autophagy in the p20-mediated NbSGS3 degradation, we silenced two autophagy-related genes (*ATGs*), *NbATG5* and *NbATG7*, to interfere with autophagy by using a tobacco rattle virus (TRV)-based virus-induced gene silencing (VIGS) assay. *NbATG5*-silenced (TRV:ATG5) and *NbATG7*-silenced (TRV:ATG7) *N. benthamiana* plants showed a similar phenotype as non-silenced (TRV:00) plants (S5A Fig), and the expression levels of *NbATG5* and *NbATG7* in silenced plants reduced to approximately 17% and 40% of that of the non-silenced plants, respectively (S5B Fig). Monodansylcadaverine (MDC)-staining revealed that the autophagy activity was significantly reduced in TRV:ATG5 and TRV:ATG7 plants compared to that in TRV:00 plants (S5C and S5D Fig). Then YFP-NbSGS3 was co-agroinfiltrated with p20-Flag or EV in TRV:ATG5, TRV:ATG7, and TRV:00 *N. benthamiana* leaves by using a half-leaf inoculation method, respectively. Immunoblotting assays showed that the p20-mediated reduction levels of YFP-NbSGS3 in leaf patches of TRV:ATG5 and TRV:ATG7 plants were lower than that in TRV:00 plants (Fig 1G). The *YFP-NbSGS3* mRNA levels did not show observable difference between EV- and p20-infiltrated patches in all plants (S4B Fig). The results indicate that the interference with autophagy activity could block the p20-mediated degradation of NbSGS3. The accumulation levels of tasiR-ARF3, an endogenous trans-acting siRNA (tasiRNA) that have been shown to be dependent on SGS3 [36], and tasiR-ARF3 target genes *N. benthamiana auxin response factor 2* (*NbARF2*), *NbARF3*, and *NbARF4* in *N. benthamiana* leaves expressing p20 and EV were measured by RT-qPCR. Results showed that the accumulation level of tasiR-ARF3 in p20-expressing leaf patches was significantly lower than that in the EV-infiltrated patches. In contrast, the accumulation levels of *NbARF2*, *NbARF3*, and *NbARF4* were significantly up-regulated in leaf patches infiltrated with p20 compared with EV (S6 Fig). Together, these results have indicated that p20 induces autophagic degradation of NbSGS3 and impairs the SGS3-dependent sRNA biogenesis.

We then examined the effect of CTV infection on the stability of NbSGS3 by co-agroinfiltrating YFP-NbSGS3 with a CTV infectious clone or infiltration buffer (Mock) in *N. benthamiana* leaves. The accumulation level of YFP-NbSGS3 was significantly lower in CTV-infected leaves compared with that in mock leaves (S7A and S7B Fig). Furthermore, the treatments of autophagy inhibitors 3-MA and E-64d enhanced the accumulation of YFP-NbSGS3 (S7C and S7D Fig), indicating that CTV infection also decreases the stability of NbSGS3 via the autophagy pathway.

### CTV p20 interacts with autophagy protein ATG8 and activates autophagy

**Fig 2:**
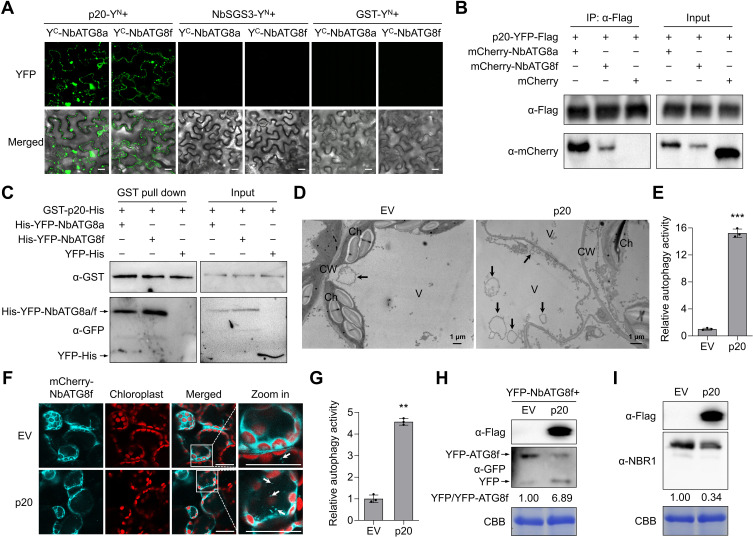
CTV p20 interacts with autophagy-related proteins NbATG8a and NbATG8f and induces autophagy. (A) Bimolecular fluorescence complementation assay for the interactions of p20 and NbSGS3 with NbATG8a and NbATG8f. The YFP fluorescence signal was detected at 60 h post-inoculation (hpi). Scale bars, 20 µm. (B) Co-immunoprecipitation assay for the interactions of p20 with NbATG8a and NbATG8f. Total protein extracts were immunoprecipitated with anti-Flag beads at 60 hpi and followed by immunoblotting using anti-Flag and anti-mCherry antibodies. (C) *In vitro* pull-down assay using GST-p20 to immunoprecipitate YFP-NbATG8a, YFP-NbATG8f, or YFP. The input and the pull-down protein samples were analyzed through immunoblotting assays. (D) Transmission electron microscopy images of autophagic structures in p20-Flag and empty vector (EV)-infiltrated *N. benthamiana* leaves. Black arrows indicate autophagic structures. Ch, chloroplast. CW, cell wall. V, vacuole. (E) Relative numbers of autophagic structures in (D). More than 20 cells were counted per experiment. (F) Confocal analysis of autophagic structures labeled by mCherry-NbATG8f in p20-Flag or EV-infiltrated leaves. The infiltrated leaves were treated with 100 µM E-64d at 48 hpi and examined at 12 h post E-64d treatment. Arrows indicate NbATG8f-labbled autophagic structures. Scale bars, 20 μm. (G) Relative numbers of autophagic structures per 15 cells in (F). More than 150 cells were counted per treatment. (H) The accumulation of free YFP released from YFP-NbATG8f upon p20 expression. Total protein was extracted at 48 hpi and then subjected to immunoblotting assay. (I) Immunoblotting assay showing the accumulation of NBR1 in p20-Flag-expressing leaves. In (E) and (G), values represent means ± SD from three independent experiments. Significant differences were identified using a one-tailed Student’s *t*-test (**, *p* < 0.01; ***, *p* < 0.001). In (H) and (I), coomassie blue staining (CBB) of the Rubisco large subunit was used as a protein loading control. The numbers below the bands represent the ratio of YFP/YFP-NbATG8f (H) and NBR1/CBB (I), respectively.

ATG8 family proteins act as autophagy receptors and adaptors to dock autophagic substrates to the autophagosomes [13]. To investigate how p20 mediates the autophagic degradation of SGS3, we examined whether p20 or SGS3 interacted with ATG8 proteins. BiFC assays showed that p20 interacted with both NbATG8a and NbATG8f, forming numerous granules in the cytoplasm (Fig 2A). In contrast, the co-expression of NbSGS3-Y^N^ with Y^C^-NbATG8a or Y^C^-NbATG8f and the negative controls failed to produce YFP signal (Fig 2A). Further luciferase complementation imaging (LCI) and Co-IP assays confirmed the interactions of p20 with NbATG8a and NbATG8f (Figs 2B and S8). GST pull-down assays showed that GST-p20 specifically bound to YFP-NbATG8a and YFP-NbATG8f, but not to the YFP control (Fig 2C). In addition, we found that p20-YFP co-localized with mCherry-NbATG8f at punctate structures in the cytoplasm (S9 Fig), which was consistent with the localization of the p20-ATG8 interaction complexes. These results indicate that p20 could localize in autophagic structures.

Since p20 interacted with NbATG8a/f, we monitored the autophagic activity in EV- and p20-infiltrated *N. benthamiana* leaves to investigate whether p20 could induce autophagy. Transmission electron microscopy (TEM) observation results showed that the p20 expression increased the number of circular autophagic structures in the vacuole compared to that in the control (Fig 2D and 2E). Consistently, the number of mCherry-NbATG8f-labeled autophagic bodies in p20-infiltrated *N. benthamiana* leaf cells increased approximately 4.6 times compared to that in EV-infiltrated cells (Fig 2F and 2G). Further MDC-staining showed that the number of autophagic structures in p20-infiltrated leaves was approximately 7.0 times higher than that in EV-infiltrated leaves (S10A and S10B Fig). YFP-ATG8f cleavage assays showed that the ratio of free YFP/YFP-NbATG8f in p20-infiltrated leaves was approximately 6.9 times higher than that in EV-infiltrated leaves (Fig 2H), and the level of an autophagy flux marker protein NBR1 in p20-infiltrated leaves was 34% of that in EV-infiltrated leaves (Fig 2I). However, the RT-qPCR results showed that the expression levels of the *ATGs NbATG1c*, *NbATG5*, *NbATG6*, *NbATG7*, *NbATG8f*, *NbATG9*, and *NbPI3K* were not significantly changed in p20-infiltrated leaves compared to that in EV-infiltrated leaves (S10C Fig), suggesting that p20-mediated autophagy activation is not strictly related to the expression levels of *ATGs*. These results indicate that CTV p20 activates autophagy in *N. benthamiana* plants.

### Autophagy plays a proviral role in CTV infection

**Fig 3:**
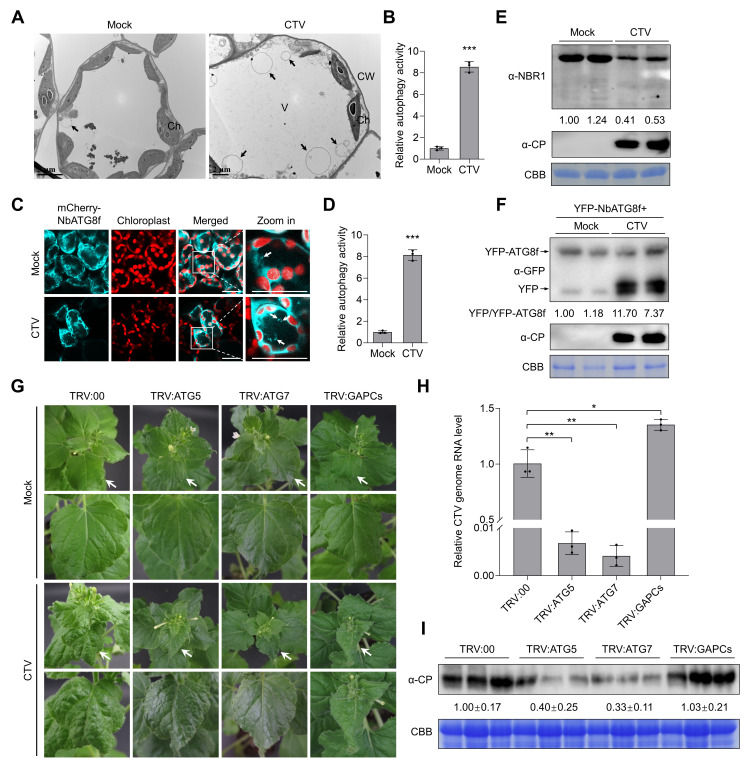
Autophagy facilitates CTV infection in *Nicotiana benthamiana.* (A) Transmission electron microscopy images of autophagic structures in mock and CTV-infected *N. benthamiana* leaves at 21 days post-inoculation (dpi). Black arrows indicate autophagic structures. Ch, chloroplast. CW, cell wall. V, vacuole. (B) Relative numbers of autophagic structures in (A). More than 20 cells were counted per treatment. (C) Confocal microscopy images of autophagic structures labeled by mCherry-NbATG8f in mock and CTV-infected *N. benthamiana* leaves at 21 dpi. Arrows indicate ATG8f-labbled autophagic structures. Scale bars, 20 μm. (D) Relative numbers of autophagic structures per 15 cells in (C). More than 150 cells were counted per treatment. (E) Immunoblotting analysis of NBR1 accumulation in mock and CTV-infected plants. Coomassie blue staining (CBB) of the Rubisco large subunit was used as a protein loading control. The numbers below the bands represent the ratio of NBR1/CBB. (F) The accumulation of free YFP released from YFP-NbATG8f in mock and CTV-infected *N. benthamiana* leaves. The numbers below the bands represent the ratio of YFP/YFP-NbATG8f. (G) Symptoms of *NbATG5*-silenced, *NbATG7*-silenced, *NbGAPCs*-co-silenced, and non-silenced plants inoculated with CTV at 28 dpi. (H, I) Relative accumulation levels of viral genomic RNA (H) and CP protein (I) in CTV-infected *NbATG5*-silenced, *NbATG7*-silenced, *NbGAPCs*-co-silenced, and non-silenced plants at 28 dpi. The band intensities of CP were calculated by ImageJ and normalized to the CBB-stained loading control. The *NbActin* gene served as an internal control in qPCR. In (B), (D), and (H), values represent means ± SD from three independent experiments. Significant differences were identified using one-tailed Student’s *t*-test (*, *p* < 0.05; **, *p* < 0.01; ***, *p* < 0.001; ns, no significance).

To investigate the effect of CTV infection on plant autophagy activity, we performed the TEM observation to examine the autophagic structures in mock- and CTV-inoculated *N. benthamiana* leaves. The circular autophagic structures were much more in CTV-infected leaf cells than that in healthy leaf cells (Fig 3A and 3B). Confocal microscope observation showed that CTV infection significantly induced the NbATG8f-labeled autophagic structures, which were approximately eight times more than that in mock-inoculated plants (Fig 3C and 3D). Further MDC-staining assays also showed that MDC-stained autophagic structures increased significantly in CTV-infected plants (about 3.9 times) (S11A and S11B Fig). Consistently, the protein accumulation of NBR1 decreased 2.4 times, and the ratio of free YFP/YFP-NbATG8f was considerably higher in CTV-infected leaves compared with that in healthy leaves (Fig 3E and 3F), indicating that CTV infection enhances autophagic flux. Moreover, the expression levels of *NbATG1c*, *NbATG5*, *NbATG6*, *NbATG8f*, *NbATG9*, and *NbPI3K* were significantly upregulated in CTV-infected plants as compared with that in mock plants (S11C Fig). These results have indicated that CTV infection induces autophagy in *N. benthamiana* plants.

We investigated the roles of autophagy in CTV infection. To determine whether autophagy affects CTV infection, the *N. benthamiana* cytosolic glyceraldehyde-3-phosphate dehydrogenase genes (*NbGAPCs*), serving as autophagy-negative regulators [37], were co-silenced as described previously to enhance autophagy (S12 Fig), and the TRV:GAPCs plants together with TRV:ATG5, TRV:ATG7, and TRV:00 plants were individually inoculated with the CTV infectious clone at 14 days post VIGS treatments. At 28 dpi, CTV infection induced severe leaf crinkle and curl symptoms in control and *NbGAPCs* co-silenced plants, but much milder symptoms occurred in *NbATG5*-silenced and *NbATG7*-silenced plants (Fig 3G). Accordingly, silencing *NbATG5* or *NbATG7* significantly decreased the accumulation levels of CTV genomic RNA and CP protein in *N. benthamiana* plants. In the *NbGAPCs* knockdown plants, CTV RNA accumulation was 1.4 times that of TRV:00 plants, but the CP level did not change (Fig 3H and 3I).

### CTV p20 links SGS3 to ATG8 for degradation

**Fig 4:**
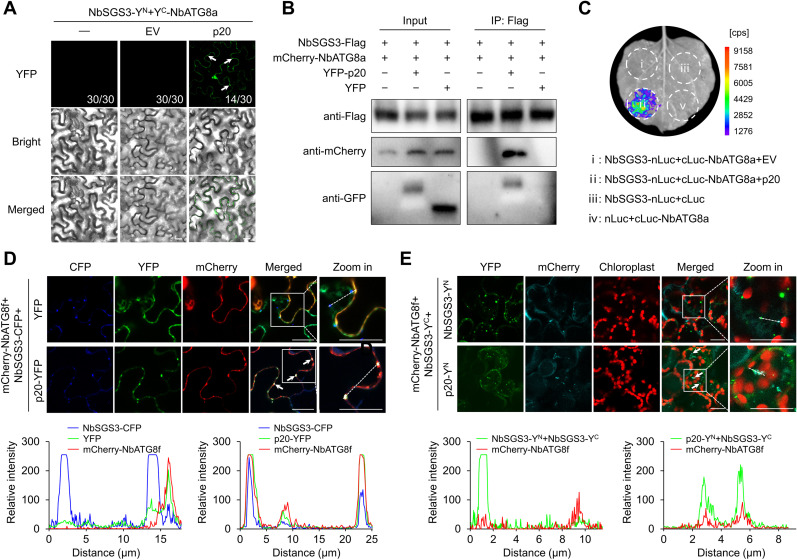
CTV p20 acts as a linker to drive SGS3 into autophagosomes. (A) Bimolecular fluorescence complementation assay of the interaction between NbSGS3 with NbATG8a with p20 as a bridge. The YFP fluorescence signal was detected at 60 h post-inoculation (hpi). Scale bars, 20 µm. (B) Co-immunoprecipitation assay showing that NbATG8a could be co-precipitated by NbSGS3 in the presence of p20. The infiltrated *Nicotiana benthamiana* leaves were treated with 100 µM E-64d at 48 hpi and harvested at 60 hpi. Total protein extracts were immunoprecipitated with anti-Flag beads and followed by immunoblotting using antibodies against Flag, mCherry, and GFP, respectively. (C) Luciferase complementation imaging assay showing the effect of p20 expression on the interaction between NbSGS3 and NbATG8a. The luciferase activity was detected at 60 hpi. The cps indicated signal counts per second. (D) Subcellular co-localization assay of p20-YFP or YFP and NbSGS3-CFP with mCherry-NbATG8f labeled autophagic bodies in *Nicotiana benthamiana* leaves. The infiltrated leaves were treated with E-64d at 48 hpi and examined at 12 h post E-64d treatment. Scale bars, 20 μm. (E) Subcellular co-localization assay of NbSGS3 self-interaction complexes or p20-NbSGS3 complexes with ATG8f-labeled autophagic bodies in *N. benthamiana* leaves. Scale bars, 20 μm. The overlapped signals in (D) and (E) were indicated by arrows. The co-localization in (D) and (E) was further analyzed by overlapping ﬂuorescence spectra, and areas indicated with white dashed lines in enlarged sections were used for this analysis.

Some autophagic substrates are sequestered into autophagosomes for degradation by the SARs [12]. Since NbSGS3 did not bind NbATG8 directly, we tested whether p20 could link NbSGS3 and NbATG8 to mediate NbSGS3 into autophagosomes. In BiFC assays, NbSGS3-Y^N^ and Y^C^-NbATG8a were co-agroinfiltrated with p20-Flag or EV into *N. benthamiana* leaves. Confocal microscope observation showed the reconstitution of YFP signals in the NbSGS3-Y^N^, Y^C^-NbATG8a, and p20-Flag co-expressing leaves but not in the leaves co-expressing NbSGS3-Y^N^, Y^C^-NbATG8a, and EV (Fig 4A). Consistently, Co-IP assays showed that NbATG8a could co-precipitate with NbSGS3 in the presence of p20 (Fig 4B). Meanwhile, in LCI assays, in contrast to the signal absence in NbSGS3-nLuc and cLuc-NbATG8a co-expressing leaves, the luminescence signals produced in NbSGS3-nLuc, cLuc-NbATG8a, and p20-Flag co-expressing leaf patches (Fig 4C).

Subcellular co-localization assays revealed that NbSGS3-CFP foci in the cytoplasm did not co-localize with NbATG8f-labeled autophagic structures (Fig 4D). At the same time, p20-YFP could promote the co-localization of NbSGS3-CFP with NbATG8f-labeled granules upon treatment with E-64d (Fig 4D), suggesting that p20 could hijack SGS3 into autophagosomes. Furthermore, the NbSGS3 self-interaction foci did not co-localize with NbATG8f-labeled autophagic structures, while the granular structures of p20-NbSGS3 complexes well co-localized with autophagic structures in the cytoplasm (Fig 4E). Thus, we conclude that CTV p20 facilitates the docking of NbSGS3 in autophagosomes.

### AIM1 and AIM5 of p20 are responsible for p20-ATG8 interaction and autophagy activation

**Fig 5:**
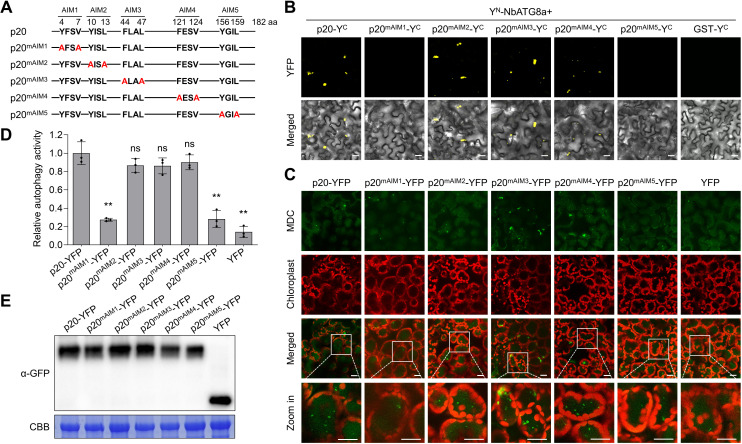
The ATG8-interacting motif 1 (AIM1) and AIM5 in p20 are indispensable for its interaction with ATG8 and autophagy induction. (A) Schematic diagram showing substitution mutants in the AIMs of p20. The AIMs in p20 were predicted using iLIR Autophagy Database (https://ilir.warwick.ac.uk/search.php). (B) Bimolecular fluorescence complementation assay showing the interactions of p20 and its substitution mutants with NbATG8a. Scale bars, 20 µm. (C) Confocal analysis of autophagy activity revealed by MDC-staining in YFP-tagged p20, YFP-tagged p20 substitution mutants, or YFP-expressing *Nicotiana benthamiana* leaves. The infiltrated leaves were treated with 100 µM E-64d at 48 h post-inoculation and examined at 12 h post E-64d treatment. Scale bars, 20 μm. (D) Relative numbers of autophagic structures per 15 cells in (C). More than 150 cells were counted per treatment. Values represent means ± SD from three independent experiments. Significant differences were identified using a one-tailed Student’s *t*-test (**, *p* < 0.01; ns, no significance). (E) Immunoblotting assay for the protein expression in (C). The Rubisco large subunit was stained with coomassie blue and used as a loading control.

Sequence analysis revealed that p20 harbored five potential ATG8-interacting motifs (AIMs) (Fig 5A). To identify the critical regions in p20 responsible for its interaction with NbATG8, we constructed five AIM substitution mutants of p20 (termed as p20^mAIM1^, p20^mAIM2^, p20^mAIM3^, p20^mAIM4^, and p20^mAIM5^) by replacing the consensus sequences ‘W/F/YxxL/I/V’ with ‘AxxA’ (Fig 5A). We found that p20^mAIM1^ and p20^mAIM5^ failed to interact with NbATG8a in BiFC assays, but all the rest mutants still interacted with NbATG8a in plants (Figs 5B). However, p20 and its mutants could interact with NbSGS3 (S13 Fig). Since p20 is an autophagy activator, we investigated whether p20-mediated autophagy induction depends on its interaction with ATG8. MDC-staining assays revealed that the autophagic structures decreased significantly in p20^mAIM1^- and p20^mAIM5^-expressing cells than that in p20-expressing cells, but the number of autophagic structures in p20^mAIM2^-, p20^mAIM3^-, and p20^mAIM4^-expressing leaves was similar to that in p20-expressing leaves (Fig 5C–5E). The results indicate that AIM1 and AIM5 are necessary for p20-ATG8 interaction and p20-mediated autophagy induction.

### The p20-ATG8 interaction is required for the VSR activity of p20

**Fig 6:**
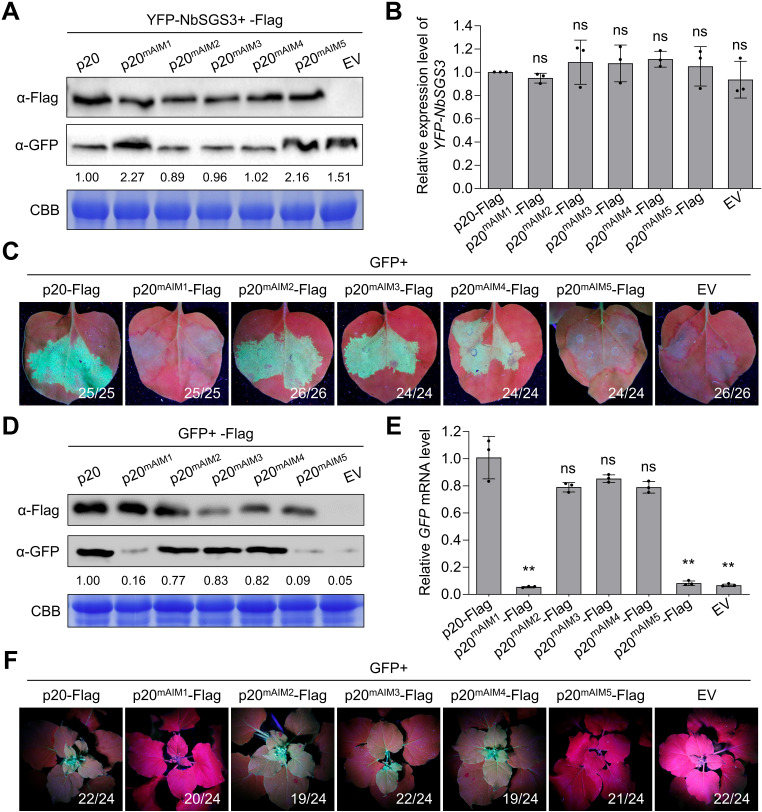
The p20-ATG8 interaction is important for the VSR activity of p20. (A) The effect of the expression of p20 and its substitution mutants on the accumulation of YFP-NbSGS3. YFP-NbSGS3 was co-agroinfiltrated with empty vector (EV), p20-Flag, or Flag-tagged p20 substitution mutants in *Nicotiana benthamiana* leaves. The accumulation levels of YFP-NbSGS3 were detected by immunoblotting assay. (B) The transcription levels of *YFP-NbSGS3* in (A) were measured by RT-qPCR assay. (C) The local RNA silencing suppressor activity of p20 and its substitution mutants. GFP was co-agroinfiltrated with p20-Flag, Flag-tagged p20 substitution mutants or EV into *N. benthamiana* 16c leaves. The GFP fluorescence signal was observed and imaged under UV light at five days post-inoculation (dpi). (D) Immunoblotting analysis of the protein expression in (C). (E) The RT-qPCR assay of the *GFP* mRNA levels in (C). (F) The systemic RNA silencing suppressor activity of p20 and its AIM substitution mutants. The GFP fluorescence signal in upper leaves was observed and imaged under UV light at 30 dpi. In (A) and (D), coomassie blue staining of the Rubisco large subunit was used as a loading control, the band intensities were calculated by ImageJ and normalized to the loading control. In (B) and (E), the *NbActin* gene served as an internal control. Values represent means ± SD from three independent experiments. Significant differences were identified using a one-tailed Student’s *t*-test (**, *p* < 0.01; ns, no significance).

We tested whether the p20-ATG8 interaction is required for p20-mediated SGS3 degradation by co-expressing NbSGS3 with p20 or its AIM mutants in *N. benthamiana* leaves. Immunoblotting assays showed that p20, p20^mAIM2^, p20^mAIM3^, and p20^mAIM4^, but not p20^mAIM1^ or p20^mAIM5^, could destabilize NbSGS3 (Fig 6A and 6B). Moreover, RT-qPCR assays showed that the expression of both p20^mAIM1^ and p20^mAIM5^ was unable to reduce the accumulation of tasiR-ARF3 or up-regulate the accumulation levels of *NbARFs* (S6 Fig). Furthermore, we individually co-expressed p20 and its AIM mutants with GFP in *N. benthamiana* 16c leaves to test the VSR activity of p20 and its mutants. At five dpi, significant GFP signals were presented in p20-Flag-, p20^mAIM2^-Flag-, p20^mAIM3^-Flag-, and p20^mAIM4^-Flag-infiltrated leaf patches. By contrast, the GFP signals disappeared in the leaf patches infiltrated with either p20^mAIM1^-Flag or p20^mAIM5^-Flag as that in EV-infiltrated patches (Fig 6C). Consistently, GFP protein and mRNA levels in leaf patches infiltrated with p20^mAIM1^-Flag and p20^mAIM5^-Flag were significantly lower than that in leaf patches infiltrated with p20-Flag and other mutants and were almost equal to that in control (Fig 6D and 6E). The systemic VSR activity of p20 and its mutants was visualized at 30 dpi. GFP signals presented in the newly developed leaves of most plants infiltrated with p20-Flag, p20^mAIM2^-Flag, p20^mAIM3^-Flag, and p20^mAIM4^-Flag, but lost in leaves of most plants infiltrated with EV, p20^mAIM1^-Flag, and p20^mAIM5^-Flag (Fig 6F). These results demonstrate that the interaction of p20 with ATG8 is critical for p20-mediated SGS3 degradation and RNA silencing suppression.

### The p20-ATG8 interaction promotes CTV infection

**Fig 7:**
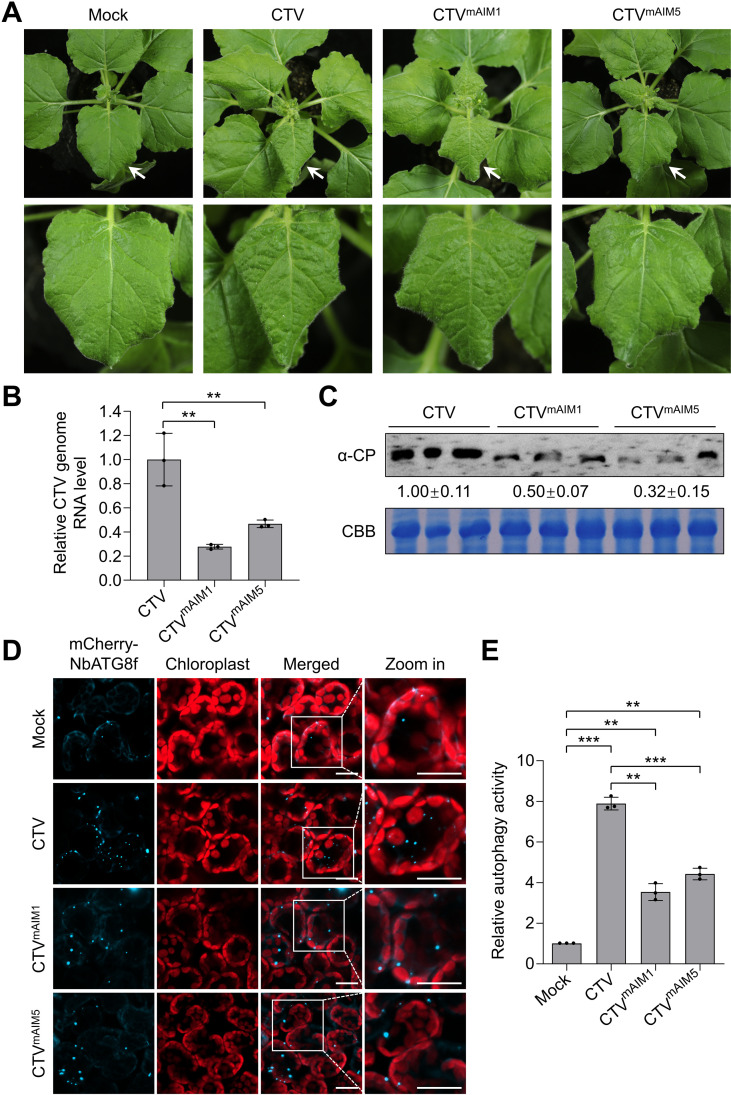
Disruption of p20 binding to ATG8 reduces CTV-induced autophagy and reduces viral infection. (A) Viral symptoms in CTV-, CTV^mAIM1^-, or CTV ^mAIM5^-infected *Nicotiana benthamiana* plants at 28 days post-inoculation (dpi). (B, C) Relative accumulation levels of viral genomic RNA (B) and CP protein (C) in CTV-, CTV^mAIM1^-, or CTV ^mAIM5^-infected plants at 28 dpi. The *NbActin* gene served as an internal control in qPCR. The band intensities of CP were calculated by ImageJ and normalized to the CBB-stained loading control. (D) Confocal analysis of autophagy activity revealed by mCherry-NbATG8f in mock, CTV-, CTV^mAIM1^-, or CTV ^mAIM5^-infected plants. The CTV-infected leaves were treated with 100 µM E-64d and examined at 12 h post E-64d treatment. Scale bars, 20 μm. (E) Relative numbers of autophagic structures per 10 cells in (D). More than 100 cells were counted per treatment. In (B) and (E), values represent means ± SD from three independent experiments. Significant differences were identified using one-tailed Student’s *t*-test (**, *p* < 0.01; ***, *p* < 0.001).

To further explore the biological importance of the p20-ATG8 interaction in CTV pathogenicity, we generated mutants CTV^mAIM1^ and CTV^mAIM5^ by replacing p20 with p20^mAIM1^ and p20^mAIM5^, respectively, in CTV infectious clone. The mutants and wild-type CTV infectious clone were individually inoculated onto *N. benthamiana* plants. At 28 dpi, the mutants CTV^mAIM1^ and CTV^mAIM5^ caused much milder leaf curling symptoms as compared to the wild-type CTV (Fig 7A). Furthermore, the accumulation levels of CTV genomic RNA and CP in plants inoculated with CTV^mAIM1^ and CTV^mAIM5^ were significantly lower than that in plants inoculated with wild-type CTV (Fig 7B and 7C). Moreover, mCherry-NbATG8f-labeled autophagic bodies in plants infected with CTV^mAIM1^ and CTV^mAIM5^ were much less than that in wild-type CTV-infected plants (Fig 7D and 7E). These results further support that the interaction of p20 with ATG8 is essential for CTV-induced autophagy and promoting the virus infection.

## Discussion

This study shows that closterovirus CTV-induced autophagy serves as a proviral mechanism during CTV infection. CTV p20 induces autophagy by interacting with ATG8 via two AIMs, AIM1 and AIM5, and relocalizes SGS3 into autophagosomes by generating the ATG8-p20-SGS3 ternary complex, finally facilitating the autophagic degradation of SGS3. These results highlight how CTV p20 takes the advantage of autophagy to impede antiviral silencing and promote CTV infection. The findings advance our understanding of the roles of autophagy in closterovirus pathogenesis.

Numerous studies have shown that VSRs repress antiviral silencing by binding siRNA and dsRNA or affecting the crucial proteins of RNA silencing [16,32,38]. Closteroviruses also encode VSRs to curb antiviral silencing [10,39]. In particular, CTV encodes three VSRs, including CP, p20, and p23, to interfere with RNA silencing in different layers [10]. Hitherto, the only study on the mechanism of closterovirus VSR is that the beet yellows virus p21 protein has sRNA-binding activity to inactivate siRNA duplexes [40,41]. In plants, vsiRNAs are generated by DCLs-mediated cleavage of dsRNAs [42]. In response, some VSRs inhibit vsiRNA accumulation by downregulating the expression level or interfering with the activity of DCLs [43,44]. It is unknown whether the closterovirus VSRs target the core proteins of RNA silencing. We found that the CTV p20 significantly reduced siRNA accumulation (S1A Fig). However, the expression of p20 did not affect the sizes and distribution profiles of *GFP* siRNAs (S1C and S1D Fig). Therefore, we hypothesized that p20 did not affect the activity of DCLs. SGS3/RDR6-dependent secondary siRNA synthesis is indispensable for RNA silencing signal amplification and systemic transduction [25,26,36]. Here, we found that p20 interacted with SGS3 and decreased the stability of SGS3 (Fig 1A–1E), suggesting that the reduced siRNA accumulation in the presence of p20 could be due to the reduced accumulation of SGS3. Consistently, p20 reduced the accumulation of SGS3-dependent endogenous tasiRNA (S6 Fig). In plant cells, SGS3 and RDR6 form heterogeneous complexes to function in the secondary siRNA synthesis [26]. However, our results showed that p20 destabilized SGS3 but not RDR6 (Figs 1D and S3). One possible explanation for this discrepancy could be that p20 hijacked the endogenous SGS3 into autophagosomes, thereby impairing the association between SGS3 and RDR6. The association of the p20 with RDR6 protein stability needs to be further studied. The viral proteins potyviral VPg, TZSV NSs, and RSMV p4 promoted the degradation of SGS3 via a combination of ubiquitination and autophagy pathways, but the molecular basis is unclear [32–34]. This study showed p20-mediated SGS3 degradation through the autophagy pathway instead of the ubiquitination pathway (Fig 1F). p20 interacted with ATG8 (Fig 2A–2C) and functioned as a scaffold to bridge SGS3 and ATG8 together, thereby hijacking SGS3 into autophagosomes (Fig 4). Consequently, our study fills the void of how a viral protein mediates the autophagic degradation of the plant antiviral element SGS3. Moreover, we found that the co-expression of p20-YN and NbSGS3-YC formed large aggregates (Fig 1A). Similarly, the potexviruses-TGBp1 co-aggregates with SGS3 to form amorphous aggregates and exhibits reduced dsRNA synthesis [45]. We speculated that the p20-induced irregular SGS3 granules also contributed to p20-mediated RNA silencing suppression.

To date, in the family *Closteroviridae*, only tomato chlorosis virus (ToCV), a species of the genus *Crinivirus*, has been shown to regulate plant autophagy [46]. However, the role of autophagy in ToCV infection remains to be characterized. In this study, we found that CTV infection and the p20 expression could induce the formation of autophagic structures and enhance autophagic flux (Figs 2D–2I and 3A–3F). Intriguingly, we found that CTV infection rather than p20 expression could upregulate the expression levels of *ATGs* in *N. benthamiana* leaves (S10C and S11C Figs). Thus, other viral factors or viral-co-opted factors might be responsible for the initiation of autophagy during CTV infection. Recent studies revealed that cotton leaf curl Multan virus βC1 and RSV CP led to the induction of autophagy by disrupting the GAPCs-ATG3 interactions [47,48]. Rhabdovirus-encoded glycoproteins trigger autophagy by promoting SnRK1-catalyzed phosphorylation of ATG6 [49]. Our results showed that two p20 mutants (p20^mAIM1^ and p20^mAIM5^) abolished their interactions with ATG8 (Fig 5B) and failed to activate autophagy (Fig 5C), indicating that the p20-ATG8 interaction is essential for p20 to induce autophagy. Furthermore, the autophagy levels in plants infected with the mutants CTV^mAIM1^ and CTV^mAIM5^ were reduced compared with that in the wild type CTV-infected plants but still much higher than that in mock plants (Fig 7D and 7E). Hence, p20 is one of autophagy activators during CTV infection and is involved in the autophagy induction at protein levels. In addition, we found that autophagy played a proviral role in CTV-plant interaction, as disrupting autophagy significantly increased plant resistance to CTV infection (Fig 3G–3I). However, the activated autophagy by silencing *NbGAPCs* only slightly increased the accumulation of viral genomic RNA (Fig 3G–3I). As CTV infection also activates autophagy, we speculate that the autophagy induction by silencing *NbGAPCs* functions redundantly with CTV-induced autophagy in promoting CTV infection. Strikingly, the mutants CTV^mAIM1^ and CTV^mAIM5^, which have a weaker ability to induce autophagy, caused milder viral symptoms and reduced viral genomic RNA and CP accumulation than wild-type CTV (Fig 7), consistent with the proviral role of autophagy during CTV infection.

Viruses counteract host SARs to escape antiviral autophagy or exploit SARs for replication or transmission [50,51]. Interestingly, a few studies have noticed that viral factors play SAR-like roles in attenuating antiviral innate responses in mammals. The matrix protein of human parainfluenza virus type 3 and the glycoprotein of Hantaan virus function as mitophagy receptors facilitating the degradation of mitochondrial antiviral signaling protein, then inhibit type I interferon response by interacting with LC3 [52,53]. Until now, whether any plant virus protein plays SAR-like roles remains to be elucidated. RSV NSvc4 triggers co-localization of plant remorins with ATG8 and the autophagic degradation of remorins, but NSvc4 cannot activate autophagy. How remorins are transported to autophagosomes in the presence of NSvc4 is undefined [22]. This study showed that p20 acted as an autophagy activator and recognized the autophagy substrate SGS3 to target its degradation (Figs 1 and 2D–2I). Significantly, p20 brought SGS3 into autophagosomes by binding ATG8 via two AIMs (AIM1 and AIM5) (Figs 4D, 4E, and 5A). These features indicate that p20 plays a SAR-like role and SGS3 is a cargo of p20. SARs recognize the substrates in a ubiquitin-dependent or ubiquitin-independent manner, and oligomerization is crucial for the function of SARs [54]. Previous studies have shown that p20 has self-interaction and SGS3 is ubiquitinated by an E3-ubiquitin ligase SGIP1 [55,56]. Further studies will determine p20 cargo proteins, the molecular basis of p20 recognizing cargo proteins and their functions in CTV infection. Recent discoveries have shown that the AIMs in tomato leaf curl Yunnan virus C1, tomato leaf curl New Delhi virus TrAP, and citrus leaf blotch virus MP are responsible for their autophagic degradation [57–59]. Considering that AIM-containing proteins can act as autophagy adaptors or receptors, exploring whether and how plant viral factors behave as SARs to target other protein degradation in plant-virus interaction would be meaningful.

**Fig 8:**
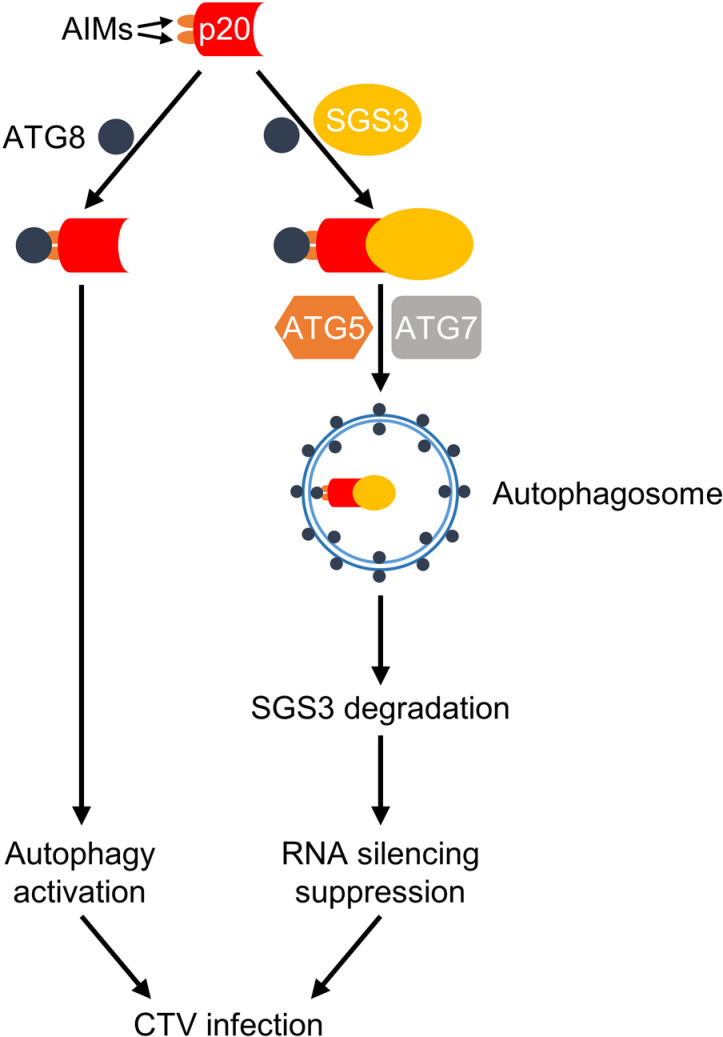
A proposed model for citrus tristeza virus (CTV) p20-mediated RNA silencing suppression. CTV-encoded p20 activates autophagy by its interaction with ATG8 through two ATG8-interacting motifs (AIMs), while the induced autophagy promotes CTV infection. Significantly, p20 serves as a bridge to link SGS3 to ATG8 to form ATG8-p20-SGS3 complexes, then the complexes are recruited into autophagosomes for degradation with the assistance of other autophagy-related proteins, including ATG5 and ATG7. Finally, the degradation of SGS3 leads to subdued antiviral RNA silencing, thus facilitating CTV infection.

In summary, we proposed a model for how p20 interferes with RNA silencing-mediated antiviral defense (Fig 8). During CTV infection, plant autophagy is induced. The p20 functions as a SAR-like factor to sequester SGS3 into autophagosomes for degradation by co-opting the autophagy adaptor ATG8 through its AIM motifs, suppressing antiviral RNA silencing and facilitating CTV infection. Our work expands the understanding of the role of autophagy in closterovirus infection and elucidates the underlying mechanism of how a viral protein utilizes autophagy to degrade host defense components for its benefits, proving that plant virus protein can also possess a SAR-like role.

## Materials and methods

### Plant materials and virus inoculation

Wild-type *N. benthamiana* and *GFP*-transgenic *N. benthamiana* 16c lines [35] were grown in a growth chamber in 60% relative humidity at 25°C with 16 h light/8 h dark photoperiod. The seeds of 16c lines were kindly donated by Prof. Xueping Zhou (Zhejiang University, Zhejiang, China). CTV isolate N21-infected *C. aurantifolia* plants were grown in an insect-proof greenhouse [60].

For CTV inoculation, *Agrobacterium* cultures (OD_600_ = 0.2) harboring the infectious clone of CTV isolate FS577 (GenBank accession no. KC517488.1) under the control of the 35S promoter, which was provided by Dr. Yan Zhou (Southwest University, Chongqing, China), were infiltrated into the leaves of four-week-old *N. benthamiana* plants with the needleless syringe. The plants infiltrated with infiltration buffer (10 mM MgCl_2_, 10 mM MES, 150 μM acetosyringone) served as negative controls. For each treatment, 10–15 plants were used, and each experiment was repeated at least three times.

### Total RNA extraction and RT-qPCR assay

Total RNA was extracted using TRIzol Reagent (Invitrogen, Carlsbad, USA). First-strand cDNA was synthesized by reverse transcription of total RNA using All-In-One RT MasterMix (with AccuRT Genomic DNA Removal Kit) (Abm, Vancouver, Canada). The expression of tasiR-ARF3 was quantified by stem-loop RT-qPCR as previously described [61]. All qPCR assays were performed in a CFX96 Real-time system (Bio-Rad, Hercules, USA) using the iTaq Universal SYBR Green Supermix (Bio-Rad, Hercules, USA) as previously described [62]. The *N. benthamiana Actin* gene (GenBank accession no. JQ256516.1) was used as an internal control. The relative expression of each gene was calculated by the cycle threshold (CT) 2^−ΔΔCT^ method [63]. The primers used for qPCR assays are listed in S1 Table.

### Small RNA-seq and data analysis

NEBNext Multiplex Small RNA Library Prep Set for Illumina (New England Biolabs, Ipswich, USA) was used to construct small RNA library. Library single-end sequencing with a 50-nucleotide reading length was performed on Illumina HiSeq2500 platform (BioMarker, Beijing, China). After removing the adaptor and low-quality reads using fastp (https://github.com/OpenGene/fastp), the obtained reads with lengths of 15 nt-30 nt were mapped to *GFP* mRNA sequence within two mismatches using bowtie2 (http://bowtie-bio.sourceforge.net/bowtie2).

### Plasmid constructions

The coding sequences of *NbSGS3* (GenBank accession no. KJ190939.1), *NbRDR6* (GenBank accession no. AY722008.1), *NbATG8a* (GenBank accession no. KX120976.1), and *NbATG8f* (GenBank accession no. KU561372.1) were amplified from a *N. benthamiana* plant. The CTV *p20* gene (GenBank accession no. OQ708955.1) was amplified from a CTV N21-infected *C. aurantifolia* plant. The mutants in the AIMs of p20 were generated by replacing the consensus amino acid sequences ‘W/F/YxxL/I/V’ of AIMs with ‘AxxA’ using an overlapping PCR method. The PCR products were purified and ligated into the pMD18-T vector (TaKaRa, Beijing, China). The sequences for these genes were confirmed by Sanger sequencing (Sangon, Shanghai, China).

For BiFC assays, the recombinant constructs were constructed by Gateway technology (Invitrogen, Carlsbad, USA). The coding sequences of p20 and NbSGS3 were individually cloned into the pEarleyGate201-YN vector [64] to fuse with the N-terminal of YN. The coding sequences of NbSGS3, p20, and p20 mutants (p20^mAIM1^, p20^mAIM2^, p20^mAIM3^, p20^mAIM4^, and p20^mAIM5^) were individually cloned into the pEarleyGate201-YC vector to fuse with the N-terminal of YC. To generated Y^N^-NbATG8a, Y^C^-NbATG8a, and Y^C^-NbATG8f, the coding sequences of NbATG8a and NbATG8f were individually recombined into the YN-pEarleyGate100 and YC-pEarleyGate100 vectors to fuse with the C-terminal of YN or YC.

The constructs used for Co-IP, subcellular localization, and VSR activity assays were constructed by using a ClonExpress II One Step Cloning Kit (Vazyme, Nanjing, China). The coding sequences of p20, p20 mutants, NbATG8f, and NbSGS3 were individually cloned into pCNF3-Flag, pCNF3-YFP-Flag, and pCNF3-CFP-HA vectors [65,66] to produce p20-Flag, p20^mAIM1^-Flag, p20^mAIM2^-Flag, p20^mAIM3^-Flag, p20^mAIM4^-Flag, p20^mAIM5^-Flag, p20-YFP-Flag, p20^mAIM1^-YFP, p20^mAIM2^-YFP, p20^mAIM3^-YFP, p20^mAIM4^-YFP, p20^mAIM5^-YFP, YFP-p20, YFP-NbATG8f, NbSGS3-Flag, YFP-NbSGS3, and NbSGS3-CFP-HA constructs. For the construction of NbRDR6-HA, NbRDR6 with a C-terminal triple HA epitope (with the stop codon) was recombined into pCNF3-Flag vector. To generate mCherry-NbATG8a and mCherry-NbATG8f, the N-terminal of NbATG8a and NbATG8f were individually fused with mCherry by overlapping PCR and cloned into pCNF3-Flag vector.

For LCI assays, the sequences encoding of p20, NbSGS3, NbATG8a, and NbATG8f were cloned into pJW771-nLUC or pJW772-cLUC vector to produce p20-nLUC, NbSGS3-nLUC, cLUC-NbATG8a, and cLUC-NbATG8f.

For pull down assays, the sequences encoding YFP and GST were amplified from pGEX-KG and pCNF3-YFP-Flag vectors, respectively, and were individually cloned into pEHIS vector to generate His-GST and His-YFP constructs. For the construction of His-GST-p20, p20 was ligated into the His-GST construct to fuse with the C-terminal of GST. To generate His-YFP-NbATG8a and His-YFP-NbATG8f, NbATG8a and NbATG8f were individually inserted into the His-YFP construct to fuse with the C-terminal of YFP.

For TRV-based VIGS, the fragments of *NbGAPC1* (GenBank accession no. KM986323), *NbGAPC2* (GenBank accession no. KM986324), *NbGAPC3* (GenBank accession no. KM986325), *NbATG5* (GenBank accession no. KX369397.1), and *NbATG7* (GenBank accession no. KX369398.1) were amplified from *N. benthamiana* and cloned into pTRV2 vector as described [37].

To generate the mutants CTV^mAIM1^ and CTV^mAIM5^, the fragment between nts 15902 and 18695 of CTV FS577 was amplified and replaced the p20 sequence with p20^mAIM1^ and p20^mAIM5^, respectively, by overlapping PCR. Then the DNA fragments were individually ligated into the infectious clone of CTV FS577 digested with *Pas*I and *Nde*I. The primers used for plasmid constructions are listed in S1 Table.

### BiFC, subcellular localization, LCI, and VSR activity assays

For BiFC and subcellular localization assays, *Agrobacterium* cultures (OD_600_ = 0.8) harboring different constructs were mixed as indicated combinations in the text in a 1:1 or 1:1:1 ratio and infiltrated into four-week-old *N. benthamiana* leaves using needle-free syringes. The fluorescence signals were detected at 48–72 h post-inoculation (hpi) by confocal laser scanning microscopy (Leica Microsystems, TCS-SP8, Germany). YFP and mCherry fluorescence was excited at 514 nm, and 561 nm and captured at 525–585 nm, and 575–630 nm, respectively. When YFP and mCherry were co-imaged, the YFP signal was excited at 514 nm and captured at 525–560 nm. The expression of proteins in the BiFC assays was confirmed by immunoblotting analysis in S2 Fig.

For LCI assays, *Agrobacterium* cultures (OD_600_ = 0.8) harboring paired cLUC and nLUC constructs were infiltrated into *N. benthamiana* leaves. At 48–60 hpi, the luciferase substrate D-luciferin solution (0.2 mM) (GlpBio, Montclair, USA) was sprayed onto the surface of infiltrated-leaves for 10–15 min and then the luciferase activities were detected using a low-light cooled CCD imaging apparatus (NightSHADE L985, Berthold, Stuttgart, Germany).

For VSR activity assays, *Agrobacterium* cultures (OD_600_ = 0.8) harboring each of the constructs were individually mixed with an equal volume of *Agrobacterium* culture (OD_600_ = 0.8) harboring 35S:GFP and then infiltrated into the leaves of four- to six-week-old *N. benthamiana* 16c plants. The empty pCNF3 vector was used as a negative control. The GFP fluorescence in infiltrated leaves was monitored at 2 dpi and 5 dpi, respectively, and the systemic VSR activity was monitored at 30 dpi under long-wavelength UV light.

### Immunoblotting and Co-IP assays

Immunoblotting and Co-IP assays were performed as previously described [67]. Total protein of collected *N. benthamiana* leaf samples was extracted with the extraction buffer (10% glycerol, 25 mM Tris, pH 7.5, 1 mM EDTA, 150 mM NaCl, 2% w/v PVPP, 10 mM DTT, 1× protease inhibitor cocktail, 0.1% NP40) at a ratio of 1:2 (w/v). The protein extracts were immunoprecipitated by anti-Flag M2 affinity gel (Sigma-Aldrich, St. Louis, USA) and incubated for 2–4 hours at 4°C. After washing the beads with IP buffer (10% glycerol, 25 mM Tris, pH 7.5, 1 mM EDTA, 150 mM NaCl, 0.1% NP40) for 5 times. The resulting protein extracts were individually solubilized in a 2 × SDS loading buffer and then separated through electrophoresis using 12% SDS-PAGE. The separated protein bands were transferred to PVDF membranes (Millipore, Billerica, MA) and detected using antibodies against Flag (ABclonal, Wuhan, China), HA (Abbkine, Wuhan, China), MYC (ABclonal, Wuhan, China), GFP (ABclonal, Wuhan, China), mCherry (Pumeike, Wuhan, China), NBR1(Abcam, Cambridge, UK), ATG8 (Abcam, Cambridge, UK), or CTV CP [68] followed with goat anti-mouse IgG-HRP secondary antibody (ABclonal, Wuhan, China) or goat anti-rabbit IgG-HRP secondary antibody (ABclonal, Wuhan, China). Blotted membranes were visualized using WesternBright ECL HRP substrate (Advansta, San Jose, USA) under the ChemiDoc Touch Imaging System.

### Pull down assay

The constructs His-GST-p20, His-GST, His-YFP-NbATG8a, His-YFP-NbATG8f, and His-YFP were individually transformed into *E. coli* Rosetta (DE3) cells. The transformed cells were grown at 37°C to OD_600_ of 0.6–0.8. Then each protein was induced with 0.5 mM isopropyl-β-D-thiogalactopyranoside (IPTG) for 12 h at 18°C. The resulting cultures were centrifuged and resuspended in ice-cold lysis buffer (0.3 M NaCl, 10 mM imidazole, 1 mM DTT, 0.25 mM PMSF, and 0.5 mg/mL lysozyme in PBS [pH 7.4]). His-tagged recombinant proteins were purified by using a high-affinity Ni-charged resin (GenScript, New Jersey, USA) as previously described [69].

Initially, we tried to express and purify NbSGS3 from *E. coli*, but failed. Instead, the YFP-tagged NbSGS3 was expressed in *N. benthamiana* leaves and precipitated by using the GFP-Trap beads (Shenzhen KT Life Technology, China). The purified GST-p20 or GST was added into the IP systems and incubated for 2 h at 4°C. To confirm the direct interaction between p20 with NbATG8a and NbATG8f, the purified GST-p20 was mixed with purified YFP-NbATG8a, YFP-NbATG8f, or YFP, followed by the addition of GST-Trap beads (GE Healthcare, USA) in PBS buffer (pH 7.4). The mixtures were incubated for 2 h at 4°C and rinsed 6 times with PBS buffer. The precipitated proteins were analyzed by immunoblotting using anti-GFP and anti-GST antibodies.

### VIGS assay

TRV-mediated VIGS assay was performed as described [70]. *Agrobacterium* cultures (OD_600_ = 0.6) harboring different pTRV2-derived constructs were individually mixed with an equal volume of *Agrobacterium* culture (OD_600_ = 0.6) harboring the pTRV1 vector, and then infiltrated into three- to four-week-old *N. benthamiana* leaves. At 14 dpi, the expression levels of target genes were measured by RT-qPCR and the phenotype of silenced plants was observed.

### Chemical treatments, MDC staining, and TEM observation

Phosphate-buffered saline (PBS) solutions containing DMSO (as control) or an equal volume of DMSO with 10 mM 3-MA, 100 μM E-64d, or 100 μM MG132 (Sigma-Aldrich, St. Louis, USA), were individually infiltrated into *N. benthamiana* leaves. After 8–12 h, the treated leaf samples were collected for further tests. MDC (Sigma-Aldrich, St. Louis, USA) staining was performed as described previously [37].

For TEM observation of autophagic structures, *N. benthamiana* leaf samples were treated with 100 μM E-64d for 8–12 h in dark and then cut into small pieces (~1 mm × 2 mm) and vacuum infiltrated with fixation solution (2.5% glutaraldehyde in 0.1 M PBS (pH 6.8)) for 12 h. After washing with PBS buffer, the samples were postfixed in 1% osmium tetroxide at room temperature for 2 h, followed by ethanol gradient dehydration and embedding in Epon 812 resin. Ultrathin sections (70 nm) were cut from embedded tissues on an Ultracut E Ultramicrotome (Reichart-Jung, Vienna, Austria) and collected on 3 mm copper (mesh) grids. The sections were double-stained with 2% lead citrate and 5% uranyl acetate before examination on a TEM apparatus (H-7000FA, Hitachi, Tokyo, Japan).

### Quantification and statistical analysis

Statistical significance was examined by one-sided Student’s *t*-test or one-way ANOVA with Tukey’s multiple-comparison test. The values for quantitative analysis were shown as means ± standard deviation (SD) from at least three independent assays. The blotted bands were quantified using ImageJ and normalized against the loading controls from the same sample.

## Supporting information

S1 Fig The effect of p20 on the composition of *GFP* siRNAs. (A) The abundance of *GFP* siRNAs in p20 or empty vector (EV)-infiltrated *Nicotiana benthamiana* 16c leaves. The Y-axis represented the proportion of *GFP* siRNAs to all sequenced sRNAs. Student’s *t*-test was used for analysis. (B) The frequency of 5’-terminal nucleotides of *GFP* siRNAs in p20 or EV-infiltrated *N. benthamiana* 16c leaves. (C) The sizes of *GFP* siRNAs in p20 or EV-infiltrated *N. benthamiana* 16c leaves. (D) Profiles of siRNAs along *GFP* mRNA in p20 or EV-infiltrated *N. benthamiana* 16c leaves. Blue and red lines indicate siRNAs from the sense and antisense of *GFP* mRNA. Values represent means ± SD (n = 3).
(TIF)

S2 Fig Immunoblotting analysis to validate the protein expression shown in Figs 1A, 2A, 4A, 4E, 5B, and S13. Coomassie blue staining (CBB) of the Rubisco large subunit was used as a protein loading control.
(TIF)

S3 Fig The effect of the p20-YFP-Flag expression on the accumulation of NbRDR6-HA in *Nicotiana benthamiana* leaves. The protein (A) and transcription (B) levels of NbRDR6-HA were measured by immunoblotting and RT-qPCR assay. Coomassie blue staining (CBB) of the Rubisco large subunit was used as a protein loading control, and the band intensities were calculated by ImageJ and normalized to the loading control. In qPCR assay, the *NbActin* gene served as an internal control. Values represent means ± SD from three independent experiments. Significant differences were identified using a one-tailed Student’s *t*-test (ns, no significance).
(TIF)

S4 Fig The transcription levels of *NbSGS3-CFP-HA* (A) and *YFP-NbSGS3* (B) in Fig 1F and Fig 1G, respectively. The *NbActin* gene served as an internal control in qPCR assay. Values represent means ± SD from three independent experiments. Significant differences were identified using a one-tailed Student’s *t*-test (ns, no significance).
(TIF)

S5 Fig TRV-based silencing of *NbATG5* and *NbATG7* in *Nicotiana benthamiana* plants. (A) The growth phenotype of *NbATG5*-silenced, *NbATG7*-silenced, and non-silenced *N. benthamiana* plants at 14 days post-inoculation (dpi). (B) Relative expression levels of *NbATG5* and *NbATG7* at 14 dpi. For RT-qPCR assay, the *NbActin* gene was used as an internal control. (C) Confocal analysis of autophagic structures labeled by MDC-staining in *NbATG5*-silenced, *NbATG7*-silenced, and non-silenced *N. benthamiana* leaves. Scale bars, 20 μm. (D) Relative numbers of autophagic structures per 15 cells in (C). More than 150 cells were counted per treatment. In (B) and (D), values represent means ± SD from three independent experiments. Significant differences were identified using a one-tailed Student’s *t*-test (*, *p* < 0.05; **, *p* < 0.01; ***, *p* < 0.001).
(TIF)

S6 Fig The expression levels of tasiR-ARF3 and tasiR-ARF3 targets in empty vector (EV)-, p20-, p20 ^mAIM1^ -, and p20 ^mAIM5^ -infiltrated *Nicotiana benthamiana* leaf patches. The *NbActin* gene served as an internal control. Values represent means ± SD from three independent experiments. Different letters indicate statistically significant differences among different groups according to the one-way ANOVA analysis with Tukey’s multiple comparison test (*p* < 0.05).
(TIF)

S7 Fig CTV infection promotes autophagic degradation of NbSGS3. (A, B) Effect of CTV infection on the stability of YFP-NbSGS3. YFP-NbSGS3 was agroinfiltrated in mock and CTV-infected *Nicotiana benthamiana* leaves. (C, D) Effect of the autophagy inhibitors and proteasome inhibitor on CTV-mediated degradation of NbSGS3. The CTV-infected *N. benthamiana* leaves were agroinfiltrated with YFP-NbSGS3 and treated with 3-MA (10 mM), E-64d (100 μM), MG132 (100 μM) or DMSO at 48 h post-inoculation and collected at 12 h after the treatments. In (A) and (C), coomassie blue staining (CBB) of the Rubisco large subunit was used as a protein loading control, and the band intensities were calculated by ImageJ and normalized to the loading control. In (B) and (D), the *NbActin* gene served as an internal control. Values represent means ± SD from three independent experiments. Significant differences were identified using one-tailed Student’s *t*-test (ns, no significance).
(TIF)

S8 Fig Luciferase complementation imaging assay for the interactions of p20 with NbATG8a and NbATG8f. The luciferase activity was detected at 60 h post-inoculation. The cps indicated signal counts per second.
(TIF)

S9 Fig Subcellular co-localization assay of mCherry-NbATG8f with YFP or p20-YFP in *Nicotiana benthamiana* leaves. The infiltrated leaves were treated with 100 µM E-64d at 48 h post-inoculation and examined at 12 h post E-64d treatment. Scale bars, 20 µm. The co-localization was further analyzed by overlapping ﬂuorescence spectra, and areas indicated with white dashed lines in enlarged sections were used for this analysis.
(TIF)

S10 Fig Effects of p20 on the MDC-stained autophagic structures and the expression levels of autophagy-related genes. (A) Confocal images of autophagic structures labeled by MDC staining in p20-Flag or empty vector (EV)-infiltrated leaves. The infiltrated leaves were treated with structures 100 µM E-64d at 48 h post-inoculation and examined at 12 h post E-64d treatment. Arrows indicate MDC-stained autophagic structures. Scale bars, 20 μm. (B) Relative numbers of autophagic structures per 15 cells in (A). More than 150 cells were counted per treatment. (C) The expression levels of autophagy-related genes in p20-Flag or EV-infiltrated *Nicotiana benthamiana* leaves. Total RNAs were extracted from infiltrated patches at three days post-inoculation. The relative expression levels of the autophagy-related gene were analyzed by RT-qPCR. The *NbActin* gene was used as an internal reference. In (B) and (C), values represent means ± SD from three independent experiments. Significant differences were identified using a one-tailed Student’s *t*-test (**, *p* < 0.01; ns, no significance).
(TIF)

S11 Fig CTV infection activates autophagy in *Nicotiana benthamiana.
* (A) Confocal analysis of autophagic structures labeled by MDC-staining in mock and CTV-infected *N. benthamiana* leaves at 21 days post-inoculation (dpi). Arrows indicate MDC-stained autophagic structures. Scale bars, 20 μm. (B) Relative numbers of autophagic structures per 15 cells in (A). More than 150 cells were counted per treatment. (C) RT-qPCR showing the relative expression levels of the autophagy-related genes in mock and CTV-infected plants at 21 dpi. The *NbActin* gene served as an internal control. In (B) and (C), values represent means ± SD from three independent experiments. Significant differences were identified using a one-tailed Student’s *t*-test (*, *p* < 0.05; **, *p* < 0.01; ***, *p* < 0.001; ns, no significance).
(TIF)

S12 Fig TRV-based silencing of *NbGAPCs* in *Nicotiana benthamiana* plants. (A) The growth phenotypes of *NbGAPCs*-silenced and non-silenced plants at 14 days post-inoculation (dpi). (B) Relative expression levels of *NbGAPCs* at 14 dpi. For RT-qPCR assay, the *NbActin* gene was used as an internal control. (C) Confocal analysis of autophagic structures labeled by MDC-staining in *NbGAPCs*-silenced and non-silenced *N. benthamiana* leaves. Scale bars, 20 μm. (D) Relative numbers of autophagic structures per 15 cells in (C). More than 150 cells were counted per treatment. In (B) and (D), values represent means ± SD from three independent experiments. Significant differences were identified using a one-tailed Student’s *t*-test (**, *p* < 0.01; ***, *p* < 0.001).
(TIF)

S13 Fig The ATG8-interacting motifs (AIMs) in p20 are dispensable for p20-NbSGS3 interaction. Bimolecular fluorescence complementation assay showing the interactions of p20 and its substitution mutants with NbSGS3. Scale bars, 20 µm.
(TIF)

S1 Table Primers used in this study. (XLSX)

S1 Data Source data for Figs 1E, 2E, 2G, 3B, 3D, 3H, 5D, 6B, 6E, 7B, 7E, S1A, S1B, S1C, S3B, S4, S5B, S5D, S6, S7B, S7D, S10B, S10C, S11B, S11C, S12B, and S12D. (XLSX)
