# Supplementary figures and images for: Citrus tristeza virus p20 suppresses antiviral RNA silencing by co-opting autophagy-related protein 8 to mediate the autophagic degradation of SGS3
Source: PLoS Pathog. 2025 Feb 24;21(2):e1012960. doi: 10.1371/journal.ppat.1012960 (PMC11882097; doi:10.1371/journal.ppat.1012960)

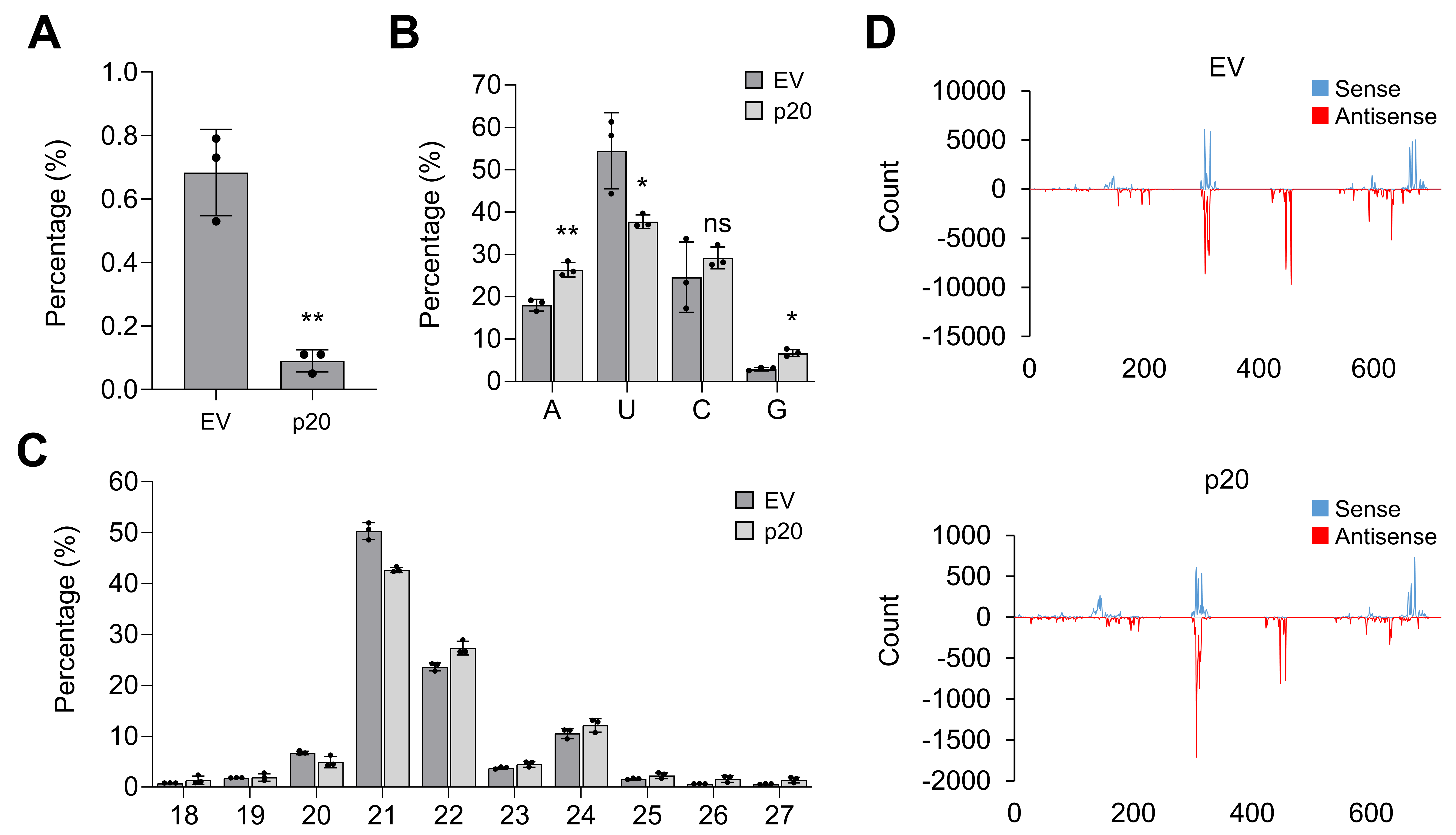

Supplement: S1 Fig — (A) The abundance of GFP siRNAs in p20 or empty vector (EV)-infiltrated Nicotiana benthamiana 16c leaves. The Y-axis represented the proportion of GFP siRNAs to all sequenced sRNAs. Student’s t-test was used for analysis. (B) The frequency of 5’-terminal nucleotides of GFP siRNAs in p20 or EV-infiltrated N. benthamiana 16c leaves. (C) The sizes of GFP siRNAs in p20 or EV-infiltrated N. benthamiana 16c leaves. (D) Profiles of siRNAs along GFP mRNA in p20 or EV-infiltrated N. benthamiana 16c leaves. Blue and red lines indicate siRNAs from the sense and antisense of GFP mRNA. Values represent means ± SD (n = 3). (TIF) [file ppat.1012960.s001.tif]

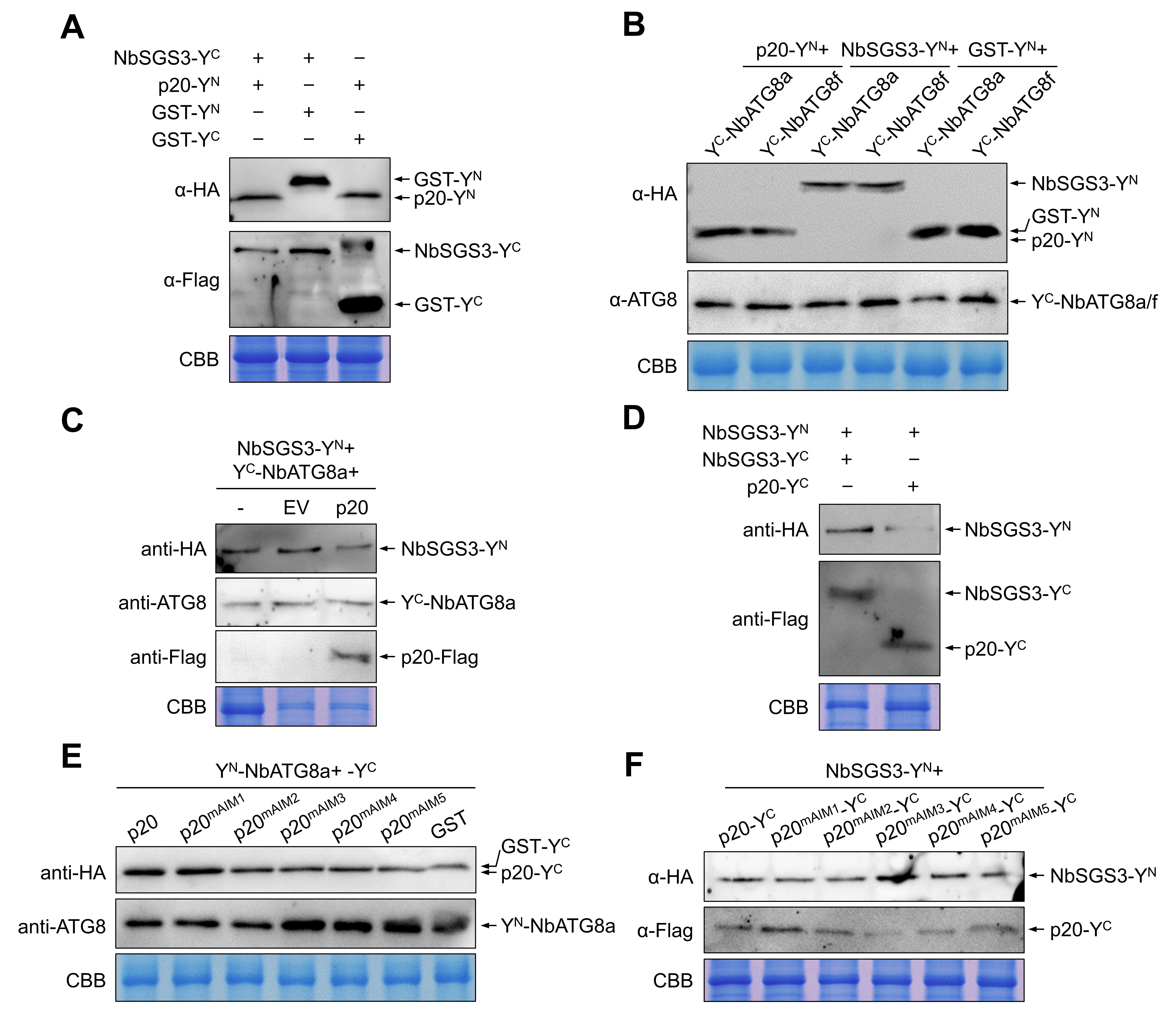

Supplement: S2 Fig — Coomassie blue staining (CBB) of the Rubisco large subunit was used as a protein loading control. (TIF) [file ppat.1012960.s002.tif]

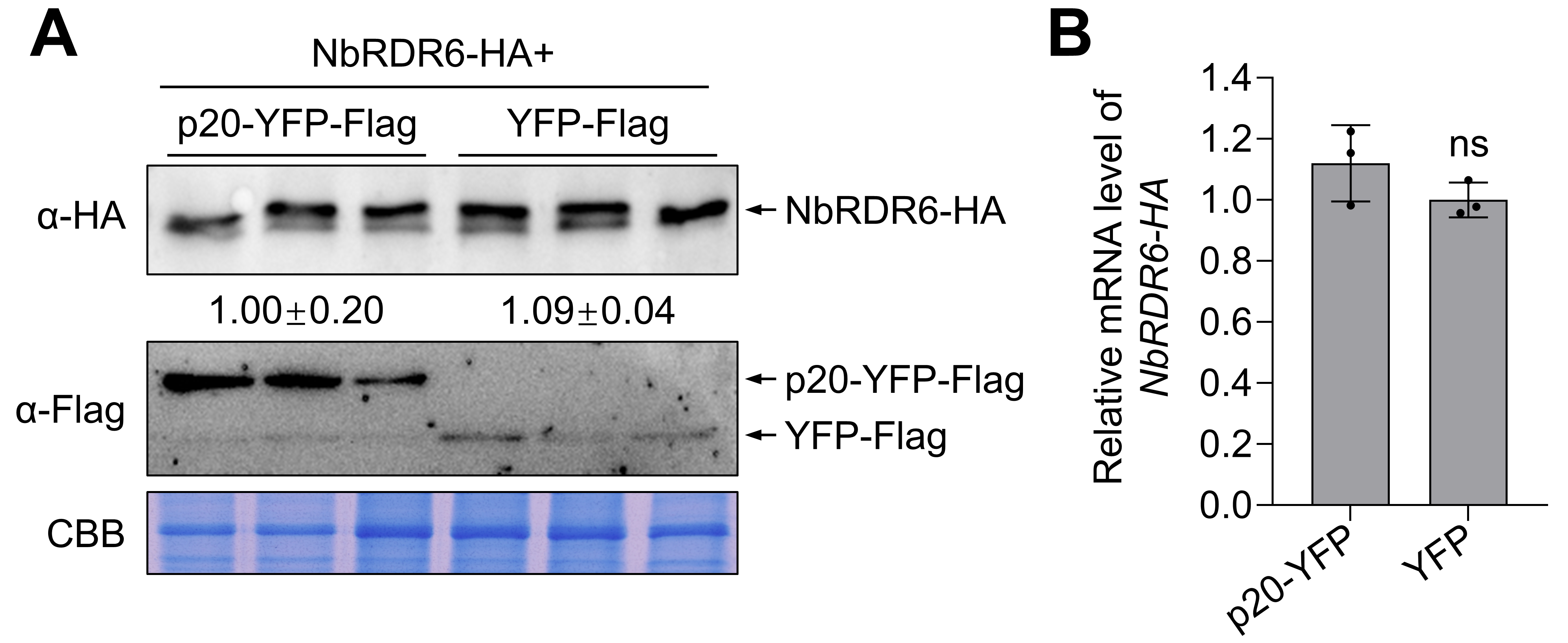

Supplement: S3 Fig — The protein (A) and transcription (B) levels of NbRDR6-HA were measured by immunoblotting and RT-qPCR assay. Coomassie blue staining (CBB) of the Rubisco large subunit was used as a protein loading control, and the band intensities were calculated by ImageJ and normalized to the loading control. In qPCR assay, the NbActin gene served as an internal control. Values represent means ± SD from three independent experiments. Significant differences were identified using a one-tailed Student’s t-test (ns, no significance). (TIF) [file ppat.1012960.s003.tif]

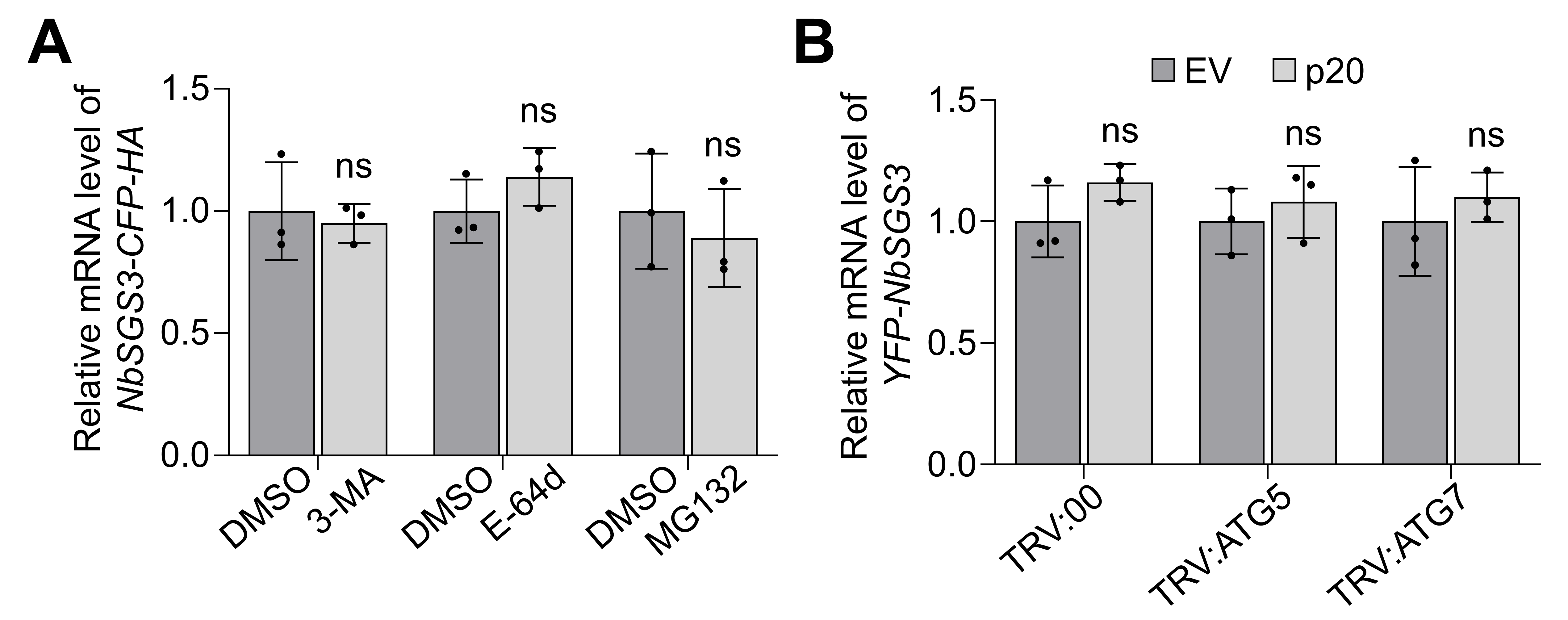

Supplement: S4 Fig — The NbActin gene served as an internal control in qPCR assay. Values represent means ± SD from three independent experiments. Significant differences were identified using a one-tailed Student’s t-test (ns, no significance). (TIF) [file ppat.1012960.s004.tif]

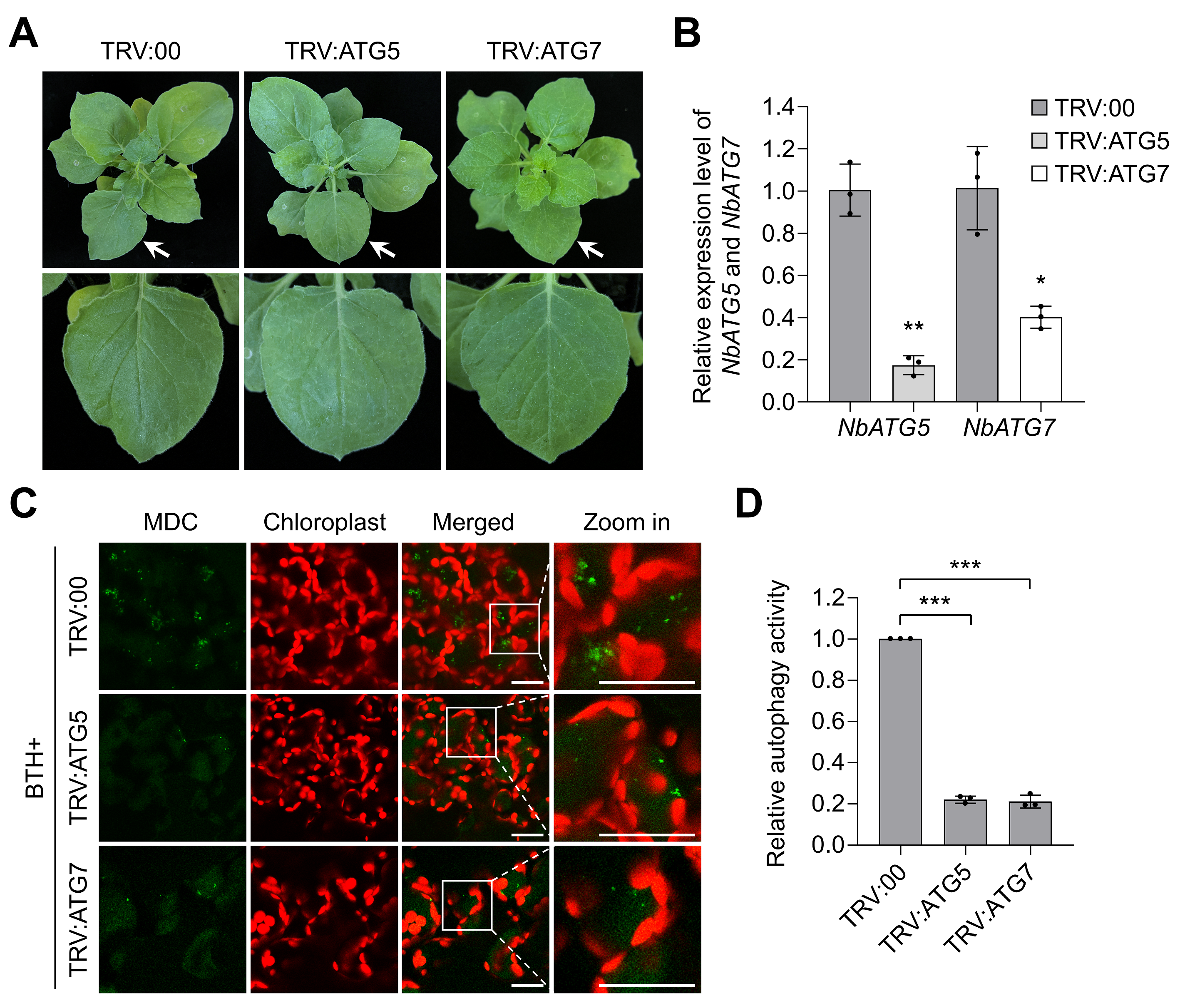

Supplement: S5 Fig — (A) The growth phenotype of NbATG5-silenced, NbATG7-silenced, and non-silenced N. benthamiana plants at 14 days post-inoculation (dpi). (B) Relative expression levels of NbATG5 and NbATG7 at 14 dpi. For RT-qPCR assay, the NbActin gene was used as an internal control. (C) Confocal analysis of autophagic structures labeled by MDC-staining in NbATG5-silenced, NbATG7-silenced, and non-silenced N. benthamiana leaves. Scale bars, 20 μm. (D) Relative numbers of autophagic structures per 15 cells in (C). More than 150 cells were counted per treatment. In (B) and (D), values represent means ± SD from three independent experiments. Significant differences were identified using a one-tailed Student’s t-test (*, p < 0.05; **, p < 0.01; ***, p < 0.001). (TIF) [file ppat.1012960.s005.tif]

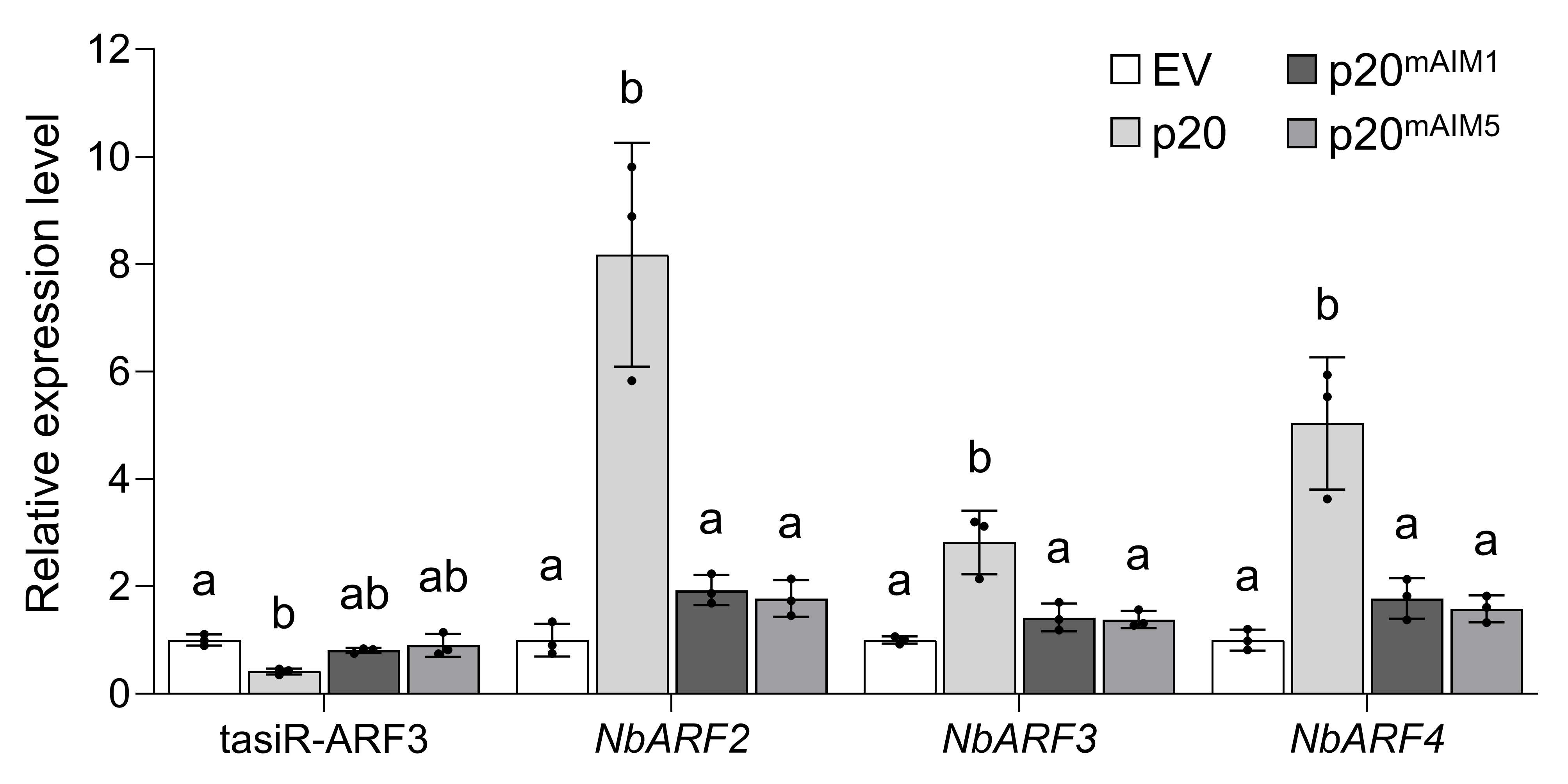

Supplement: S6 Fig — The NbActin gene served as an internal control. Values represent means ± SD from three independent experiments. Different letters indicate statistically significant differences among different groups according to the one-way ANOVA analysis with Tukey’s multiple comparison test (p < 0.05). (TIF) [file ppat.1012960.s006.tif]

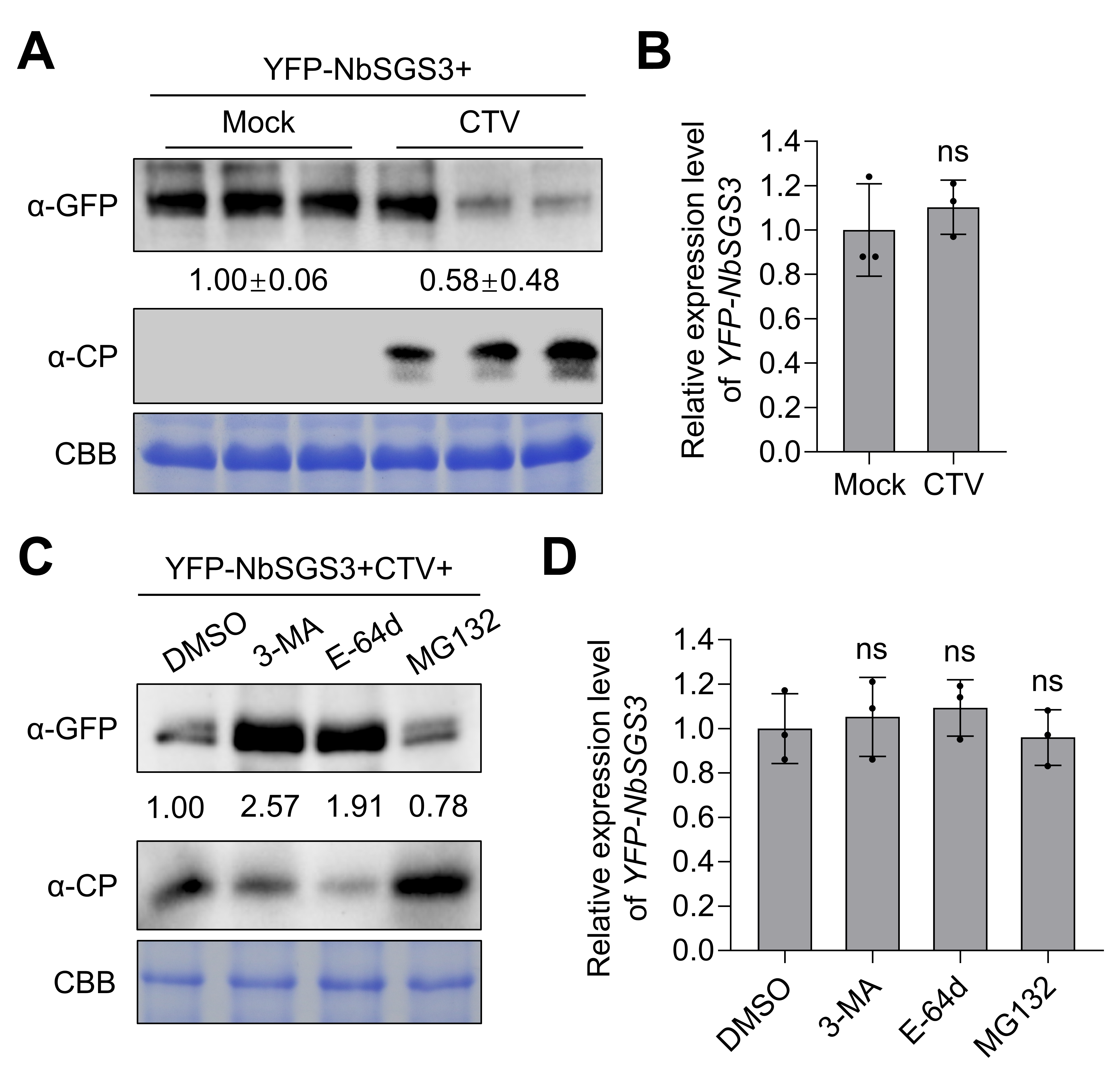

Supplement: S7 Fig — (A, B) Effect of CTV infection on the stability of YFP-NbSGS3. YFP-NbSGS3 was agroinfiltrated in mock and CTV-infected Nicotiana benthamiana leaves. (C, D) Effect of the autophagy inhibitors and proteasome inhibitor on CTV-mediated degradation of NbSGS3. The CTV-infected N. benthamiana leaves were agroinfiltrated with YFP-NbSGS3 and treated with 3-MA (10 mM), E-64d (100 μM), MG132 (100 μM) or DMSO at 48 h post-inoculation and collected at 12 h after the treatments. In (A) and (C), coomassie blue staining (CBB) of the Rubisco large subunit was used as a protein loading control, and the band intensities were calculated by ImageJ and normalized to the loading control. In (B) and (D), the NbActin gene served as an internal control. Values represent means ± SD from three independent experiments. Significant differences were identified using one-tailed Student’s t-test (ns, no significance). (TIF) [file ppat.1012960.s007.tif]

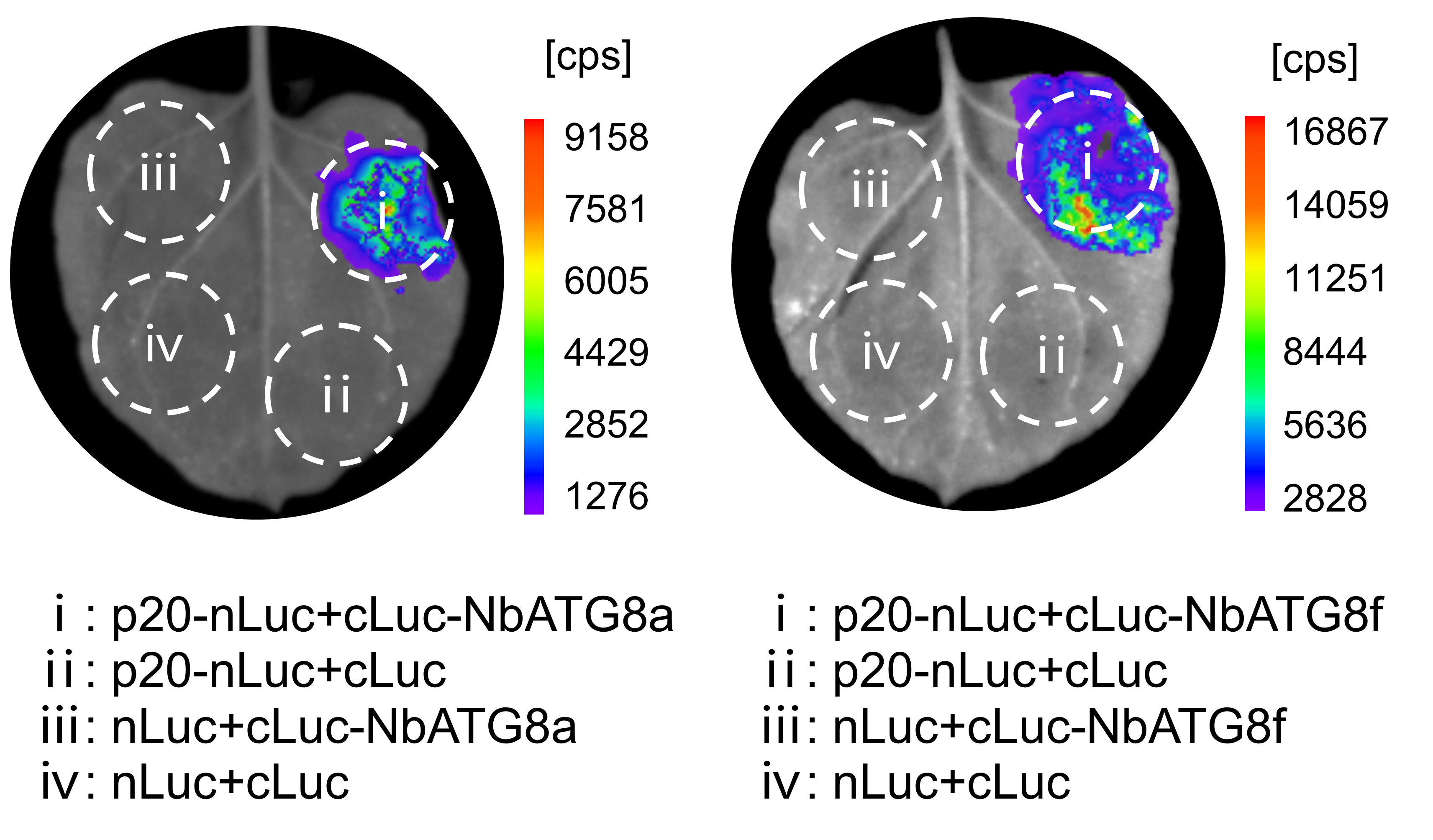

Supplement: S8 Fig — The luciferase activity was detected at 60 h post-inoculation. The cps indicated signal counts per second. (TIF) [file ppat.1012960.s008.tif]

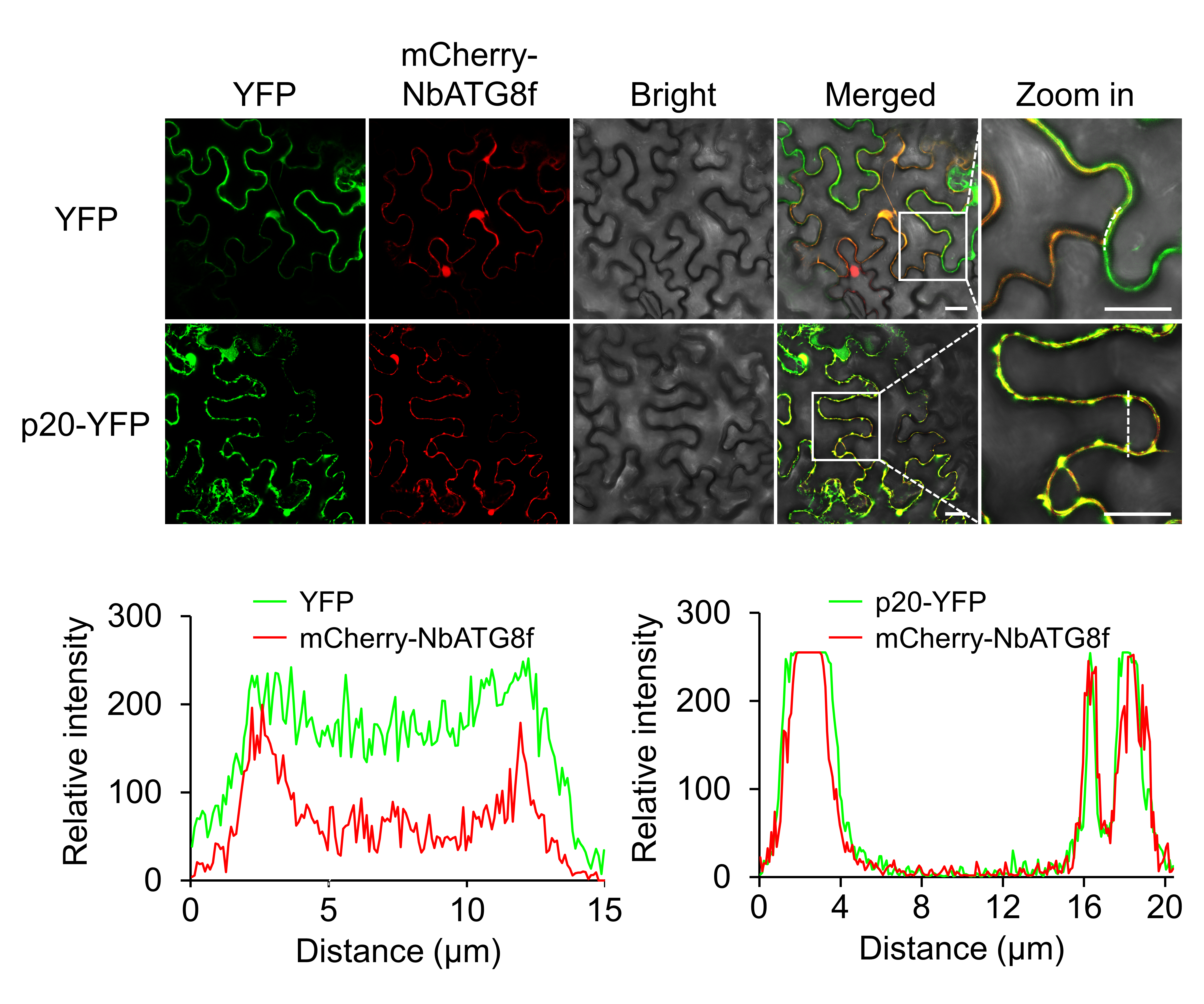

Supplement: S9 Fig — The infiltrated leaves were treated with 100 µM E-64d at 48 h post-inoculation and examined at 12 h post E-64d treatment. Scale bars, 20 µm. The co-localization was further analyzed by overlapping ﬂuorescence spectra, and areas indicated with white dashed lines in enlarged sections were used for this analysis. (TIF) [file ppat.1012960.s009.tif]

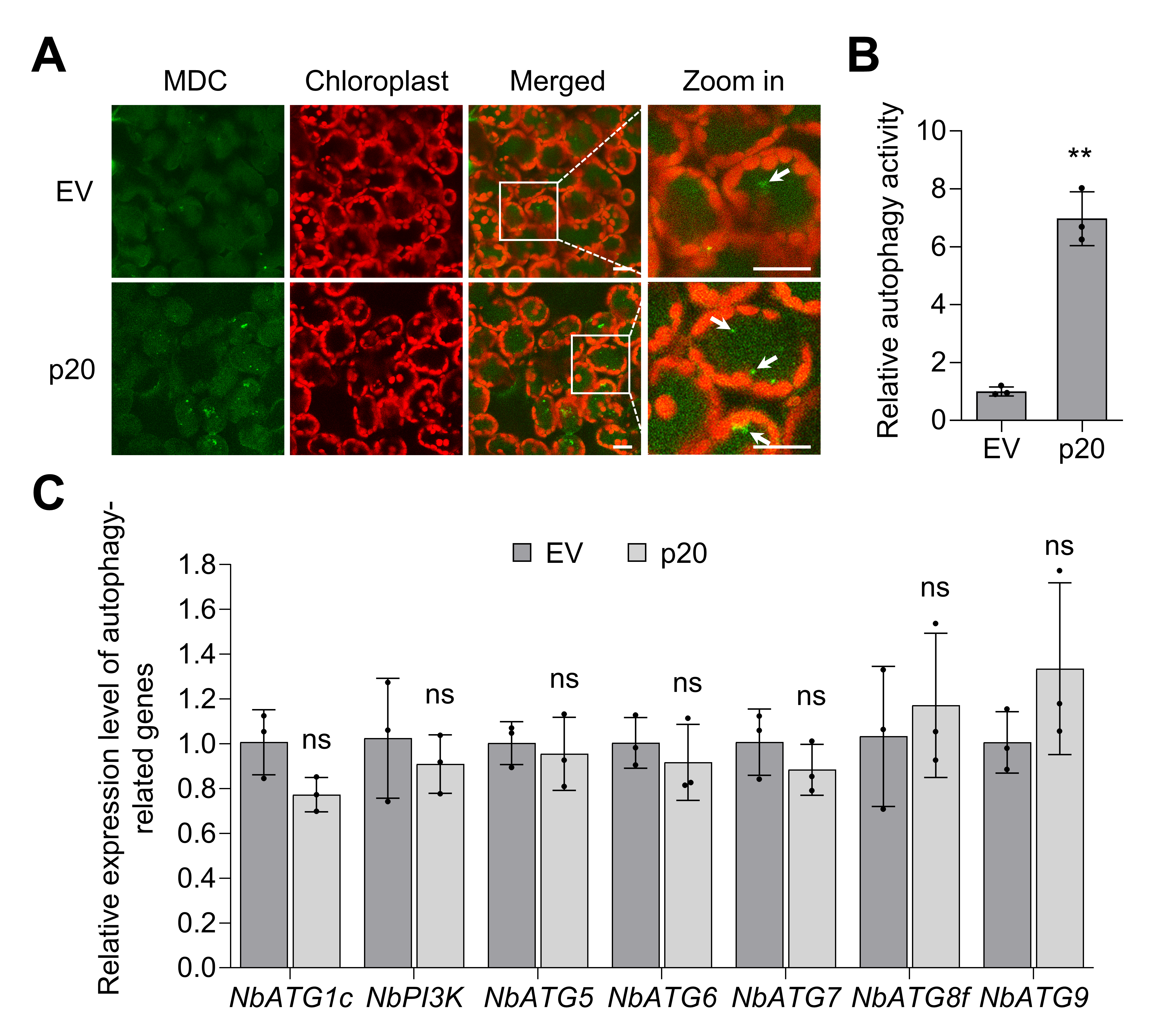

Supplement: S10 Fig — (A) Confocal images of autophagic structures labeled by MDC staining in p20-Flag or empty vector (EV)-infiltrated leaves. The infiltrated leaves were treated with structures 100 µM E-64d at 48 h post-inoculation and examined at 12 h post E-64d treatment. Arrows indicate MDC-stained autophagic structures. Scale bars, 20 μm. (B) Relative numbers of autophagic structures per 15 cells in (A). More than 150 cells were counted per treatment. (C) The expression levels of autophagy-related genes in p20-Flag or EV-infiltrated Nicotiana benthamiana leaves. Total RNAs were extracted from infiltrated patches at three days post-inoculation. The relative expression levels of the autophagy-related gene were analyzed by RT-qPCR. The NbActin gene was used as an internal reference. In (B) and (C), values represent means ± SD from three independent experiments. Significant differences were identified using a one-tailed Student’s t-test (**, p < 0.01; ns, no significance). (TIF) [file ppat.1012960.s010.tif]

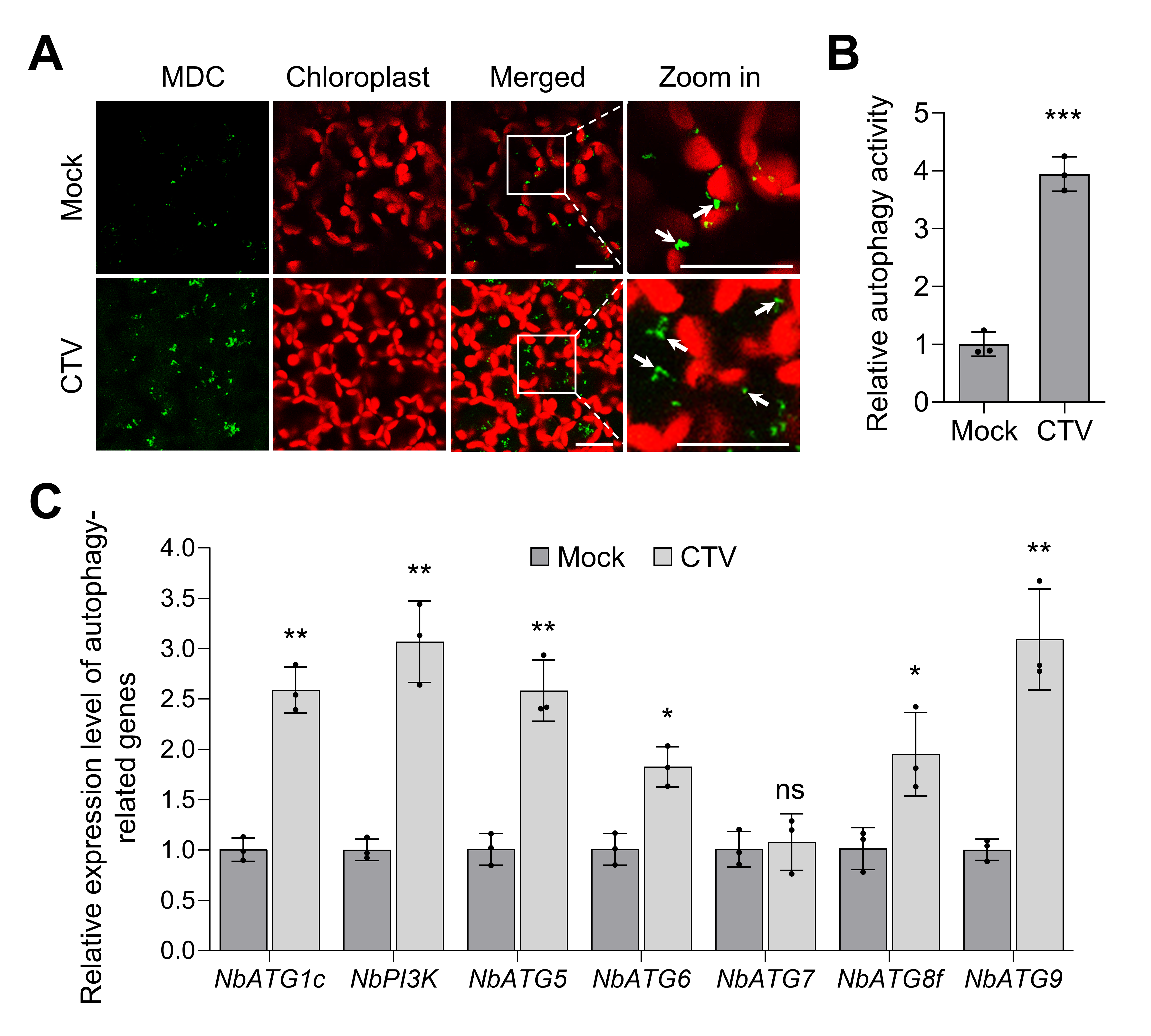

Supplement: S11 Fig — (A) Confocal analysis of autophagic structures labeled by MDC-staining in mock and CTV-infected N. benthamiana leaves at 21 days post-inoculation (dpi). Arrows indicate MDC-stained autophagic structures. Scale bars, 20 μm. (B) Relative numbers of autophagic structures per 15 cells in (A). More than 150 cells were counted per treatment. (C) RT-qPCR showing the relative expression levels of the autophagy-related genes in mock and CTV-infected plants at 21 dpi. The NbActin gene served as an internal control. In (B) and (C), values represent means ± SD from three independent experiments. Significant differences were identified using a one-tailed Student’s t-test (*, p < 0.05; **, p < 0.01; ***, p < 0.001; ns, no significance). (TIF) [file ppat.1012960.s011.tif]

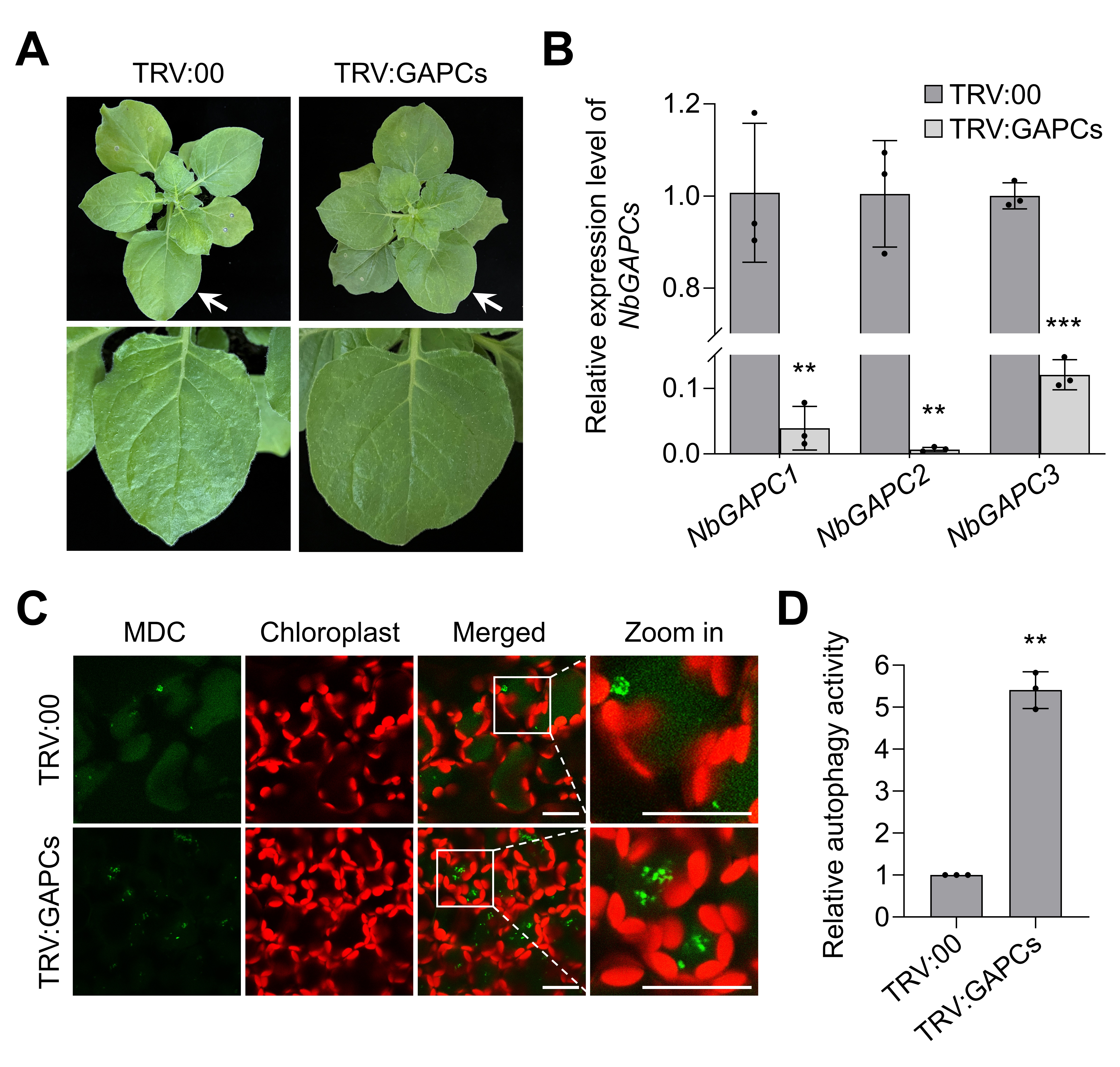

Supplement: S12 Fig — (A) The growth phenotypes of NbGAPCs-silenced and non-silenced plants at 14 days post-inoculation (dpi). (B) Relative expression levels of NbGAPCs at 14 dpi. For RT-qPCR assay, the NbActin gene was used as an internal control. (C) Confocal analysis of autophagic structures labeled by MDC-staining in NbGAPCs-silenced and non-silenced N. benthamiana leaves. Scale bars, 20 μm. (D) Relative numbers of autophagic structures per 15 cells in (C). More than 150 cells were counted per treatment. In (B) and (D), values represent means ± SD from three independent experiments. Significant differences were identified using a one-tailed Student’s t-test (**, p < 0.01; ***, p < 0.001). (TIF) [file ppat.1012960.s012.tif]

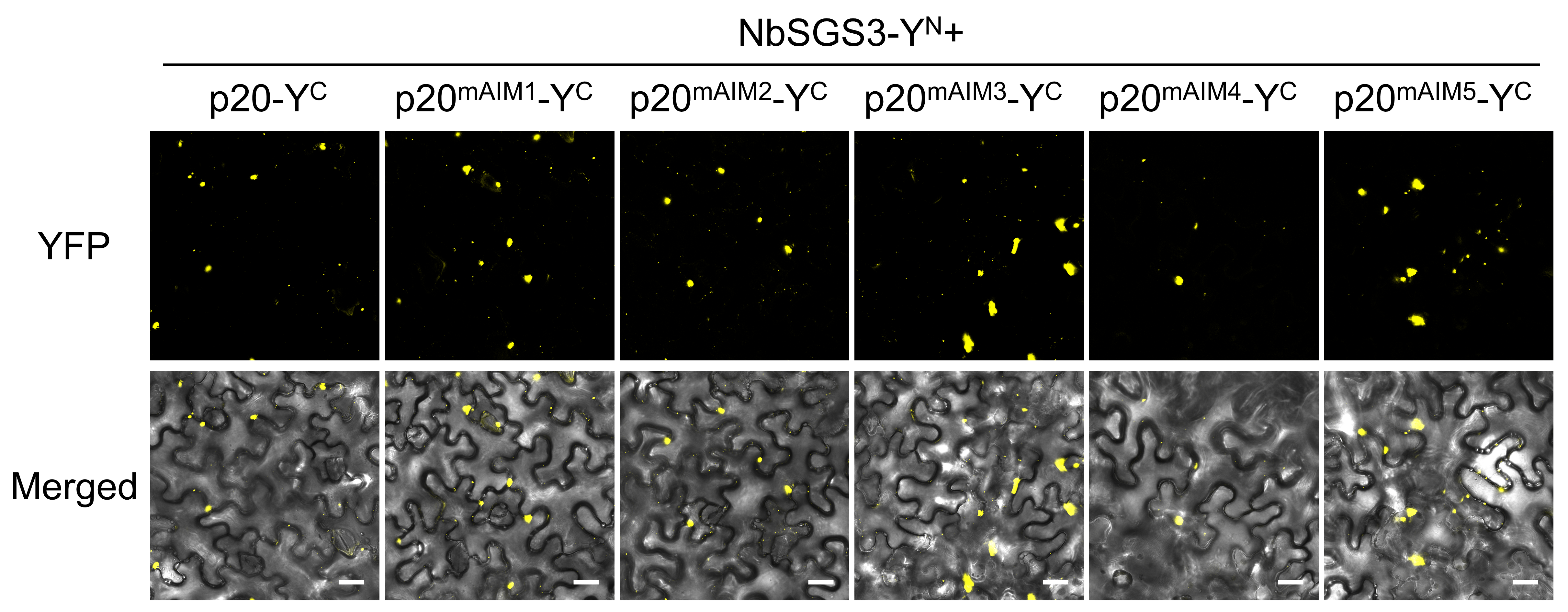

Supplement: S13 Fig — Bimolecular fluorescence complementation assay showing the interactions of p20 and its substitution mutants with NbSGS3. Scale bars, 20 µm. (TIF) [file ppat.1012960.s013.tif]
